# A New Strategy for Deleting Animal drugs from Traditional Chinese Medicines based on Modified Yimusake Formula

**DOI:** 10.1038/s41598-017-01613-7

**Published:** 2017-05-04

**Authors:** Jinghui Wang, Yan Li, Yinfeng Yang, Xuetong Chen, Jian Du, Qiusheng Zheng, Zongsuo Liang, Yonghua Wang

**Affiliations:** 1 Key Laboratory of Xinjiang Endemic Phytomedicine Resources, Pharmacy School, Shihezi University, Ministry of Education, Shihezi, 832002 China; 2 Key laboratory of Industrial Ecology and Environmental Engineering (MOE), Faculty of Chemical, Environmental and Biological Science and Technology, Dalian University of Technology, Dalian, 116024 China; 3 College of Life Science of Zhejiang Sci-Tech University, Hangzhou, 310018 China

## Abstract

Traditional Chinese medicine (TCM), such as Uyghur Medicine (UM) has been used in clinical treatment for many years. TCM is featured as multiple targets and complex mechanisms of action, which is normally a combination of medicinal herbs and sometimes even contains certain rare animal medicinal ingredients. A question arises as to whether these animal materials can be removed replaced from TCM applications due to their valuable rare resources or animal ethics. Here, we select a classical UM Yimusake formula, which contains 3 animal drugs and other 8 herbs, and has got wealthy experience and remarkable achievements in treating erectile dysfunction (ED) in China. The active components, drug targets and therapeutic mechanisms have been comprehensively analyzed by systems-pharmacology methods. Additionally, to validate the inhibitory effects of all candidate compounds on their related targets, *in vitro* experiments, computational analysis and molecular dynamics simulations were performed. The results show that the modified, original and three animal materials display very similar mechanisms for an effective treatment of ED, indicating that it is quite possible to remove these three animal drugs from the original formula while still keep its efficiency. This work provides a new attempt for deleting animal materials from TCM, which should be important for optimization of traditional medicines.

## Introduction

Traditional Chinese medicine (TCM) including Uyghur Medicine (UM) has been used for pharmaceutical and dietary therapy for several millennia^1^. UM is also characterized as multiple chemical components and multiple targets, which has been acknowledged with remarkable curative effects in treating some complex diseases such as arthritis, rheumatism, chronic back leg pain^2^. Due to these extensive use and the remarkable therapeutic effects, the interest in studying UM is gradually increased nowadays.

However, UM often contains not only, as usual as common TCMs, a combination of many medicinal herbs, but also certain animal medicinal materials^3^. Some of these animal taxa like rhino, tiger, or musk beer, etc., are now at the brim of extinction due to commercial overexploitation^4^. Additionally, poaching of animals for their medicinal uses has also brought many wild species under the red data book, for a possibility of their extinction. For instance, bear bile has a decades-long history in the treatment of hepatic and biliary disorders, but it is just due to this extensive consumption of bear bile that has made bears become endangered species^5^. Moreover, the rapid deterioration of the natural biodiversity, as well as the loss and destruction of the habitat have also caused many rare animals in danger of extinction^4^. Due to these reasons, animal species for therapeutic purposes are becoming more and more precious, a question of whether animal ingredients in UM preparations can be removed arises, due to the current limitations of experimental approaches and the complex function mechanism of “multiple components, multiple targets” of TCM systems.

In this work, we have selected a well-known UM Yimusake formula, which is commonly used not only by Uyghur medical doctors for treating erectile dysfunction (ED) but also by ordinary people for self-medication^6^. This formula contains 11 medicinal species, of which three are animal drugs, i.e., *Moschus*, *Ambra Grisea* and *Bullwhip*. Among the three animal ingredients, *Moschus* (Shexiang in Chinese), the glandular secretion of male musk deer, has many pharmacological activities including the resuscitation, activating blood to promote menstruation, detumescence and analgesia, which has been applied in about 10% commonly used Chinese formulated products^7^. Owing to the expense and wide applications of *Moschus*, the population of musk deer has actually steeply declined to the verge of extinction^8^. As to *Ambra Grisea*, it is a faecal product of the sperm whale (Physeter catodon), which has long been prized in eastern practice as medicine, condiment or perfume^9^. It shows the effects of curing migraine headaches, common colds, constipation and improving sexual performance^10^. For the third animal material, *Bullwhip*, it is the external genital organs of male cattle, which exhibits property of warming and tonifing kidney-Yang effects^11^. In the present study, we choose Yimusake formula to explore the possibility to modify the formula by removing its animal materials while still remaining its curative effects by systems-based methods.

Due to the fact that herbal formulae with numerous chemical compounds are too complex to be examined solely by reduction theory-based approaches, the mechanisms of action for most UM are poorly understood. The trend of investigating traditional medicines has been transferred to systems analysis with the emergence of the TCM systems pharmacology for elucidation of the bioactive components and mechanisms of action^12–16^. In this work, we use a systems-pharmacology screening platform which integrates multiple methods including the text mining, ADME screening, target identification, and network analysis to build the drug-target networks for uncovering the therapeutic mechanisms of this formula. The ligand-binding assays are in good agreement with the observed drug-target network indicating that the drug-target interactions obtained are reliable. The results show that it is quite possible to remove the three animal components, i.e., the *Moschus*, *Ambra Grisea* and *Bullwhip materials*, from this traditional medicine without endangering its original pharmaceutical effects. Hopefully, this work can provide a strategy to modify traditional Chinese medicines by removing/replacing those animal ingredients, and in this way to facilitate the TCM drug discovery and modernization as well.

## Materials and Methods

### Herb Data Mining

To obtain herbs for the treatment of ED, a robust wide-scale text mining of PubMed and the clinical trial database was carried out with the keywords ‘herbal medicine’ and ‘erectile dysfunction’. Owning to diverse herbs with different research extents, *P-value* was employed to equilibrate this bias and further appraise the chance probability of co-occurrence of each herb and ED^17^.

1 $$P=1-\sum _{i=1}^{k-1}f(i)=1-\sum _{i=0}^{k-1}\frac{(\begin{array}{c}K\\ i\end{array})(\begin{array}{c}N-K\\ n-i\end{array})}{(\begin{array}{c}N\\ n\end{array})}$$

where *N* is the total number of papers published in PubMed and CNKI, *K* is the number of articles linked with ED, *n* is the number of articles about one single herb and *k* is the number of articles about the effects of corresponding herbs on ED. Here, when *P-value* is less than 0.01, the herbal medicine is regarded as having significant correlation with ED. In addition, more empirically based knowledge and TCM experience are employed for the selection of herbs.

Presently, we take the Yimusake formula as a probe to modify the original herbal preparations. In detail, all animal drugs, i.e. *Moschus*, *Ambra Grisea*, *bullwhip*, and one toxic herb-*Papaveris Pericarpium* are removed. Additionally, since *Gymnadenia Conopsea* has medicinal effects similar to that of *Tuber Salep*, we use *Gymnadenia Conopsea* replace *Tuber Salep* in terms of clinical use^11^. In final, we got a modified formula of Yimusake established by using the combinations of *Gymnadenia Conopsea*, *Stigma Croci*, *Semen Strychni*, *Boswellia*, *Semena Myristica*, *Syringa Oblata* and *Rhizoma Alpiniae Officinarum*.

### Molecular Database

All compounds of these herbs from the modified Yimusake formula were collected from our TCMSP database (http://lsp.nwu.edu.cn). TCMSP comprises 510 herbal entries registered in Chinese pharmacopoeia with more than 33,000 ingredients. It supports simple and advanced web-based query options that can specify the searching clauses, such as the molecular properties, structures, TCM ingredients and 2D/3D visualization of the molecules, targets and diseases. Considering the fact that glycosides could be hydrolyzed to their aglycone forms before being absorbed, in this section, their corresponding aglycones were also added. Finally, a total of 619 chemicals are included in the present analysis.

### ADME Screening

An early assessment of the absorption, distribution, metabolism, and excretion (ADME) properties of drug candidates has become an essential process in modern drug discovery. Proper utilization of ADME results, meanwhile, can prioritize those candidates that are more likely to have good pharmacokinetic properties and also minimize the potential drug-drug interactions. In the current work, two ADME-related models, including PreOB (predict oral bioavailability) and PreDL (predict drug-likeness) were employed to prescreen the bioactive molecules.

Oral bioavailability (%F) is considered a key parameter during the drug development. Oral drug absorption is determined by two fundamental parameters: the drug’s solubility and its gastrointestinal permeability^18^. Presently, the PreOB model, developed on the basis of a robust in-house system OBioavail 1.1^19^, was performed to predict the OB of the constituents of the herbs in the formula. The molecule with suitable OB ≥30% was chosen as candidate compound for further research.

Drug-likeness is an established concept in drug discovery that aims to identify virtual or real molecules that fall into what is considered to be drug-like chemical space, based on one or more physicochemical properties^20^. In this study, we have used 3,206 experimental drugs for developing a knowledge-based computational model for predicting the drug-likeness of a molecule. Compounds with DL ≥0.18 were selected as the candidate bioactive molecules, because the mean value of DL for all 3,206 molecules in DrugBank (http://www.drugbank.ca/) is 0.18. The molecular data were all uploaded to TCMSP (http://lsp.nwsuaf.edu.cn/tcmsp.php).

### Target Identification and Network Construction

Identification of protein targets for bioactive small molecules has been a crucial step for drug repositioning and drug discovery. Predicting drug-target interactions could help understand the underlying biological mechanisms from the network perspective. Presently, to predict the potential drug targets, an in-house software: SysDT^21^ was employed. SysDT is a method developed on the basis of Random Forest and Support Vector Machine, which incorporates a large scale of chemical, genomic and pharmacological data^21^.

In addition, to visualize and analyze the drug-target interactions, a group of compounds and their potential targets were used for the construction of compound-target (C-T) networks. All of the candidate chemicals are obtained from ADME screening and the targets are derived from the target fishing. The bipartite graphs are generated by Cytoscape 2.8.1^22^, an open source of bioinformatics package for biological network analysis and visualization.

### Experimental Validation

To validate the accuracy and efficiency of the C-T networks, we constructed *in vitro* experiments to further validate the inhibitory effects of compounds on their predicted targets. The ligand-binding assays were performed to quantify the inhibitory effects of drugs on their predicted direct targets according to the manufacturer’s instructions. Two key and commercially available targets were selected on the market. Targets F2 (Thrombin Inhibitor Screening Kit) and MAOB (Monoamine Oxidase B Inhibitor Screening Kit) were purchased from BioVision. The purity of the compounds methyllinolenate, quercetin, curcumin, isorhamnetin, galangin, macelignan, chrysin, eugenol, kaempferol in each sample was higher than 98% which compounds were all purchased from YuanYe Technology Ltd. (Shanghai, China). In order to avoid the loss of activity under long-term storage, these drugs were dissolved in DMSO and freshly prepared.

### Preparation of Computational Analysis

For exploring the mechanism of the binding modes and offering more insights into the interactions between the candidate compounds and their protein targets, eleven C-T interactions from the ligand-binding assays of experimental validation were selected for docking simulations. The computational modeling of protein-ligand complexes were carried out by using molecular docking with GOLD version 5.1, a genetic algorithm-based docking program to generate an ensemble of docked conformations. Taking into account the factors including H-bonding energy, van der Waals energy, metal interaction, and ligand torsion strain in the defaulted scoring function, the GOLD Score fitness function was employed^23^. The X-ray crystal structures of F2 and MAOB (PDB entry codes: 2R2M and 2V61) were retrieved from RCSB Protein Data Bank. Finally, the optimal conformation of each compound was selected for further analysis by assessing the score and the interactions between ligands and target.

### Molecular Dynamics Simulations

In order to explore the interaction and illustrate the accurate binding model for the active site of F2 and MAOB with their ligands, molecular dynamics (MD) simulations were carried out using the GROMACS software package^24^. The 3D structures of the 11 docked complexes with ligands were analyzed in detail and served as the starting structure for MD simulations. Using the GROMOS 96 force field^25^ combined with a periodic boundary condition that used the particle mesh Ewald method^26^ and a normal pressure and temperature ensemble at 300 K, the calculation was performed. The temperature was maintained constant by the Berendsen thermostat, the value of the isothermal compressibility was set to 4.5 × 10^−5^ bar^−1^ while the pressure was maintained at 1 bar using the Parrinello-Rahman scheme^27^.The cut-off distances for the calculation of Coulomb and van der Waals interactions were 1.0 and 1.4 nm, respectively. Prior to the simulation, energy minimization was performed for the full system without constraints using the steepest descent integrator. The system was equilibrated via 500 ps MD simulations and all the simulations lasted for 5 ns ensure the stability of the whole system.

### Drug-Pathway Interaction

To examine the systematic effects of herbal compounds on disease, an integrated “ED-related pathway” was assembled based on the following steps: (1) the obtained target profiles were organized into several pathways by mapping to KEGG; (2) the pathways not directly related to ED were removed according to pathological and clinical studies; and (3) the ED-related pathway was manually synthesized, containing the nitric oxide (NO)/cyclic guano-sine monophosphate (cGMP) pathway, Ras kinase pathway and Vascular endothelial growth factor (VEGF) pathway.

## Results

Over recent years, herbal medicine as a complementary and alternative therapy for the treatment of ED has become increasingly popular^28^. Many factors may play roles in the pathophysiology of ED, in which three ones are major causes of it, i.e., the vascular, neurologic and hormonal abnormalities. Therefore, in this study, we focus on these three aspects to explore the mechanism of action of the different constituents in this formula on the pathophysiology of ED.

Traditionally, *Stigma Croci* is believed to attribute to vascular ailments, *Semen Strychni* shows therapeutic effects on central nervous system (CNS) disorders, *Boswellia*, *Syringa Oblata* and *Rhizoma Alpiniae Officinarum* can treat hormonal abnormalities. In addition, *Gymnadenia Conopsea* is also effective on vascular diseases and disorders induced by hormone, while *Semena Myristica* can be used for treating vascular- and CNS-linked aliments. Here, by systems pharmacology-based methods, we focus on exploring the mechanism of action of these constituents in this formula on the pathophysiology of ED.

### Potential Active Compounds

Due to the fact that TCMs are often orally administered, the analysis of their ingredients’ oral bioavailability dependent on the absorption, distribution and liver metabolism conditions *in vivo*, is crucial for finding out those active compounds in an herb. Additionally, to remove those compounds that seem to be chemically unsuitable for drugs, the drug-likeness property based on the Tanimoto coefficient is introduced. In this section, PreOB and PreDL models were used to screen out the ingredients of the modified Yimusake formula with favorable pharmacokinetic properties. All the chemicals meeting with the filtering criteria: OB ≥30% and DL ≥0.18 are considered as candidate compounds.

### Stigma Croci

*Stigma Croci* is a spice derived from the flower of the saffron crocus^29^. It has a wide use against a variety of diseases in China and other Asian countries. Specifically, male subjects always use *Stigma Croci* for the prevention of premature ejaculation or infertility and women use it for premenstrual syndrome or menstrual cramps^30^. It is also used for genitourinary system-related diseases including abortion, amenorrhea, aphrodisiac, impotency, contraceptive, emmenagogue, prolapse of anus, stop menstrual periods, promote menstruation, painful urination, diuretic and kidney stone^31^. In addition, *Stigma Croci* can also increase the bioavailability and enhance the absorption of other drugs^32^.

In the present study, 6 compounds were obtained with favorable OB and DL from this herb. For examples, crocetin has antioxidant activity for male factor infertility^33^. Crocetin can also increase libido, enhance erectile function, and ameliorate semen quality^34^. This validates the reasonability of our prescreening model.

Besides, in animal models, safranal and picrocrocin increased sexual behavioral factors such as mounting frequency, intromission frequency and erection frequency^31^. In humans, saffron (200 mg/day, 10 days) showed significant aphrodisiac activity on sexual function, accompanying with the increased numbers and durations of erectile events in patients with ED^31^. In addition, safranal demonstrated a positive effect on semen parameters in terms of sperm motility in those men suffering from idiopathic infertility^34^. Therefore, these compounds are also added for further analysis, irrespective of their low DL values.

### Semen Strychni

*Semen Strychni* has been used as folk medicine for alleviating inflammation, improving blood circulation, joint pains and allergic symptoms with a long history^35^. It is also used as aphrodisiac, appetizer, digestive, purgative, and stimulant^36^. In this herb, up to date 13 compounds have been identified from the crude nux vomica. Among these, alkaloids have been proved to be the main bioactive components responsible for the analgesic, anti-oxidant, anti-tumor, anti-inflammatory activity^37^. Strychnine and brucine N-oxide are the major alkaloids found in *Semen Strychni*. Earlier studies have shown that these alkaloids were responsible for their various pharmacological effects^38^. Among them, strychnine has been employed clinically for nervous system disease^38^, vomiting and traumatic pain^39^. With respect to brucine N-oxide, it showed strong effects in the inhibition of prostaglandin synthesis^40^, since the abdominal writhing induced by acetic acid involves the process or the release of arachidonic acid metabolite via cyclooxy-genase and prostaglandin biosynthesis. Brucine N-oxide also reduced the vascular permeability through the reduction of leakage into the peritoneum^40^.

In addition to the alkaloids mentioned above, another one is brucine, an odorless white crystalline solid alkaloid (molecular weight, 394.45), which is also the second most abundant alkaloid in the *Semen strychnine*. Although brucine shows relatively weak OB values (OB = 7.6%), the data suggests that it exerted following pharmacological effects: cough suppressant, microcirculation facilitation, cell protection, pain relief, anti-rheumatic and anti-tumor effects^41^. Also, brucine inhibited VEGF-induced cell proliferation, chemotactic motility, and the formation of capillary-like structures in HUVECs in a dose-dependent manner^42^. Owing to these profound pharmacological effects, brucine was also selected for further research as well.

### Gymnadenia Conopsea

*Gymnadenia conopsea*, an orchidaceae perennial plant, is widely distributed in the northern parts of China such as Hebei, Liaoning, and Gansu Provinces. The tubers of this plant have long been used for the treatment of asthma, neurasthenia, chronic hepatitis and male factor infertility^43^. Here, 11 compounds were obtained with good OB and favorable DL values from this herb. Surprisingly, most of these compounds have been proven to possess various biological activities^44^. For example, six ones, i.e., two dihydrophenanthrenes, gymconopins A (M005) and gymconopins B (M001) together with known phenanthrene and stilbene constituents, 1-(4-hydroxybenzyl)-4-methoxyphenanthrene-2,7-diol(M004), 1-(4-hydroxybenzyl)-4-metho-xy-9,10-dihydrophenanthrene-2,7-diol(M002), 3,3′-dihydroxy-2-(4-hydroxybenzyl)-5-me-thoxybibenzyl(M003), 3′,5-dihydroxy-2-(4-hydroxybenzyl)-3-methoxybibenzyl (M007), were found to inhibit the antigen-induced degranulation by 65.5 to 99.4% at 100 mM in RBL-2H3 cells^40^. Among these, Gymconopin A and gymconopin B were used as aphrodisiacs for the treatment of sexual dysfunction^45^. In addition, Gymnosides II had radical scavenging activities for DPPH radical and O^−^_2_ effects, which helpfully maintained the healthy arteries and blood vessels^46^.

Although glucosyloxybenzyl 2-isobutylmalates dactylorhin A, dactylorhin B, loroglossin and militarine have low OB, these four glucosyloxybenzyl 2-isobutylmalates exhibited significant biological activities. For instance, dactylorhin A exerted antioxidative mechanism on vascular endothelial cells^47^. Antioxidants preserve fatty acids from oxidation, and thus, may play an important role in male fertility^48^. Dactylorhin B also has the activity of reducing the toxic effects of b-amyloid fragment on neuron cells^47^. Therefore, as supplement to our predicted results, these constituents were also treated as active ingredients.

### Boswellia

*Boswellia*, known as Shallaki Guggal, grows widely on dry hills of the Indian states of Madhya Pradesh, Bihar and Gujarat^49^. As a famous crude drug in TCM, *Boswellia* has been used to treat rheumatic arthralgia, chest obstruction, dysmenorrhea, mouth sores, ringworm, diarrhea, vaginal discharge, hemorrhoids, hair loss, fever, dysentery, skin, blood, and syphilitic diseases, amenorrhea and infertility^50, 51^. Pharmacological research has demonstrated that *Boswellia* possesses analgesic, anti-inflammatory, sedative, antihyperlipidemic, antibacterial, carminative, mental tonic, eye tonic, anthelmintic, stomachic, antiulcer, and antipyretic activities^52^.

Presently, a total of 9 compounds with good OB and DL values are obtained (Tale1), most of which have been reported as bioactive ingredients. For example, boswellic acids, the principal active constituents of *Boswellia*, have been shown to possess diverse activities including antitumor, spasmolytic, antiviral, hepatoprotective, gastro-protective, antidiabetes, antimicrobial, hemolytic, anti-inflammatory, antipruritic and antithrombotic^53^. Hussain, H. *et al*. reported that boswellic acid inhibited the proliferation of MCF-7 cells substantially through the regulation of VEGF, and the oral administration of this drug increased the fertility of rats^51^. By acting on the pituitary gland, boswellic acid increased main hormones of spermatogenesis, showing a proerectile effect in rabbits^54^. Additionally, 11-keto-β -boswellic acid, one of the potent anti-inflammatory compounds of *Boswellia*, inhibited the androgen receptors by the interference of the Sp1-binding activity for treating in hormone-related diseases^55^. The previous studies in both humans and experimental models have confirmed various bioactivities of 11-keto-β-boswellic acid, such as anti-inflammatory, anticancer, antioxidant, antiulcer and antibacterial effects^56^. Presently, all these 9 potential bioactive compounds are analyzed for further target prediction in this herb.

### Semena Myristica

*Semena Myristica*, commonly known as nutmeg, is an evergreen aromatic tree cultivated in many tropical countries^57^. Due to the presence of sterols, phenols, alkaloids and amino acids in this herb, it has been mentioned in Unani medicine to be of value in the management of male sexual disorders^58^. Additionally, *Semena Myristica* has been reported to have aphrodisiac, carminative, stomachic, tonic, astringent, aromatic, ntithrombotic, aantifungal, sedative and memory-enhancing, anti-inflammatory, antioxidant activities^58, 59^. Moreover, the effects of *Semena Myristica* on increaseing blood circulation, improving the mounting behavior and mating performance of male Swiss mice have also been demonstrated^60, 61^. In this herb, 9 potential compounds are found meeting the screening criteria and plenty of them with satisfactory ADME properties have already been shown exerting significant protective effects against several diseases. For instance, beta-sitosterol, one of the main active constituents of *Semena Myristica*, has been clinically proven to play a role in male fertility^62^. Through modulation of antioxidant enzymes on endothelial cells, beta-sitosterol shows binding affinity for the estrogen receptors and protects against oxidative stress^63^.

Moreover, some compounds with relatively poor pharmacokinetic properties were also collected as the active components for the following study due to their confirmed biological activities. For example, although myristicin in *Semena Myristica* has low OB (18%) and DL (0.07), it has been reported possessing aphrodisiac activity and increasing both the libido and potency, which might be attributed to its nervous and hormonal stimulating property^58^. Besides, myristicin has been reported to exhibit multiple pharmacological activities such as antioxidant, antimicrobial, hepatoprotective, antidepressants, anxiogenic, anti-HIV and anticancer activities^64^. Thus this molecule is also retained for further analysis.

### Syringa Oblata

Among the popular ornamental bushes, *Syringa Oblata* is cultivated in the middle latitudes of Eurasia and North America^65^. Owing to the highest antioxidative activities of *Syringa Oblata*, it is associated with many diseases, including male fertility, cancer, arteriosclerosis, diabetes, and immune deficiency^66^. In addition, *Syringa Oblata*, as a safe and inexpensive source of natural antioxidants, its crude extracts and/or pure compounds are raw materials for the preparation of health supplements^67^.

For this herb, 7 potential bioactive compounds are obtained for further target prediction, most of which have been proven possessing various biological activities. For example, stigmasterol, as an important constituent of *Syringa Oblata*, showed pharmacological activities such as antioxidant, anti-osteoarthritic, anti-hypercholestrolemic, cytotoxicity, antitumor, hypoglycaemic, anti-mutagenic and anti-inflammatory and CNS effects^68^. Since stigmasterol also participated in the synthesis of many hormones^68^, it can act as a precursor in the synthesis of progesterone and an intermediate in the biosynthesis of androgens, estrogens and corticoids, showing a positive effect on male fertility^69^. What’s more, numerous preclinical studies have confirmed that another active compound kaempferol has a wide range of pharmacological aspects such as antioxidant, antidiabetic, neuroprotective, cardioprotective, antimicrobial, anticancer, anti-inflammatory, anti-osteoporotic, estrogenic/antiestrogenic, anti-allergic, anxiolytic and analgesic activities^70^.

### Rhizoma Alpiniae Officinarum

*Rhizoma Alpiniae Officinarum*, the dry root and rhizome of *Alpinia officinarum Hance*, is a TCM mainly distributed in southern China^71^. With strong antioxidative activities, *Rhizoma Alpiniae Officinarum* has been demonstrated as an effect replacement for R-tocopherol^72^. In recent years, more and more biological activities of *Rhizoma Alpiniae Officinarum* have been reported, including anti-coagulation, anti-diabetic, anti-bacterial, anti-ulcer, anti-diarrhea, anti-emetic, analgesia, anti-tumor, anti-fungal, anti-inflammatory properties^72, 73^.

The obtained result shows that 15 ingredients are screened out, possessing not only satisfactory OB, but also favorable DL. Interestingly, most constituents of *Rhizoma Alpiniae Officinarum* have been reported to show profound pharmacological effects. For instance, galangin, as one of the major flavonoids in *Rhizoma Alpiniae Officinarum*, has been demonstrated various biological activities including anti-oxidative, anti-clastogenic, anti-mutagenic, metabolic enzyme modulating and free radical scavenging activity^74, 75^. Besides, it can inhibit the proliferation of human mammary tumor cells by down-regulation of cyclins D3, E and A^74^. Pinocembrin, another major flavonoid molecule from *Rhizoma Alpiniae Officinarum*, exhibited estrogenic activity with EC_50_ value of 67 μM^75^. It has a large range of pharmacological activities including antioxidant, anti-inflammatory, antimicrobial and anticancer activities^76^. Moreover, pinocembrin can be used as neuroprotective against cerebral ischemic injury with a wide therapeutic time window, which might be useful for treatment of diseases in the CNS^77^. Therefore, these candidate compounds exert biological activities in the treatment of ED and are used for further target prediction.

Taken together, a total of 66 candidate compounds (Table 1) are obtained from the OB, DL, and drug metabolism prescreening *in vivo*.

**Table 1:** Bioactive compounds of the modified Yimusake formula with their OB and DL values predicted.

ID	Compound	OB	DL	Herb Name
M001	gymconopin B	44.85	0.54	*Gymnadenia Conopsea*
M002	1-(4-hydroxybenzyl)-4-methoxy-9,10-dihydrophenanthrene-2,7-diol	44.08	0.55	*Gymnadenia Conopsea*
M003	3,3′-dihydroxy-2-(4-hydroxybenzyl)-5-methoxybibenzyl	43.80	0.38	*Gymnadenia Conopsea*
M004	1-(4-hydroxybenzyl)-4-methoxyphenanthrene-2,7-diol	38.97	0.55	*Gymnadenia Conopsea*
M005	gymconopin A	38.69	0.38	*Gymnadenia Conopsea*
M006	gymnosides II	35.24	0.59	*Gymnadenia Conopsea*
M007	3′,5-dihydroxy-2-(4-hydroxybenzyl)-3-methoxybibenzyl	34.42	0.38	*Gymnadenia Conopsea*
M008	2-methoxy-9,10-dihydrophenanthrene-4,5-diol	33.31	0.18	*Gymnadenia Conopsea*
M009	coelovirin A	31.07	0.59	*Gymnadenia Conopsea*
M010	coelovirin B	31.07	0.60	*Gymnadenia Conopsea*
M011	coelovirin E	4.33	0.23	*Gymnadenia Conopsea*
M012	n-heptanal	79.74	0.59	*Stigma Croci*
M013	isorhamnetin	49.60	0.31	*Stigma Croci*, *Rhizoma Alpiniae Officinarum*
M014	quercetin	46.43	0.28	*Stigma Croci*, *Syringa Oblata*
M015	crocetin	35.30	0.26	*Stigma Croci*
M016	kaempferol	41.88	0.24	*Stigma Croci*, *Syringa Oblata, Rhizoma Alpiniae Officinarum*
M017	safranal	39.56	0.04	*Stigma Croci*
M018	picrocrocin_qt ^a^	33.71	0.04	*Stigma Croci*
M019	methyllinolenate	46.15	0.18	*Stigma Croci*
M020	beta-sitosterol	36.91	0.75	*Myristica Semena*, *Syringa oblata*, *Rhizoma Alpiniae Officinarum*
M021	galbacin	61.00	0.53	*Myristica Semena*
M022	licarin B	53.11	0.40	*Myristica Semena*
M023	cis-permethrin	45.06	0.38	*Myristica Semena*
M024	saucernetindiol	41.85	0.32	*Myristica Semena*
M025	isonectandrin B	62.86	0.32	*Myristica Semena*
M026	threo-austrobailignan-5	49.49	0.32	*Myristica Semena*
M027	isoguaiacin	48.78	0.31	*Myristica Semena*
M028	dihydroguaiaretic acid	31.32	0.26	*Myristica Semena*
M029	macelignan	23.60﻿﻿﻿	0.32﻿﻿	*Myristica Semena*
M030	myristicin	17.99	0.07	*Myristica Semena*
M031	(S)-stylopine	51.15	0.85	*Semen Strychni*
M032	isostrychnine N-oxide (II)	37.33	0.80	*Semen Strychni*
M033	isostrychnine N-oxide (I)	35.45	0.80	*Semen Strychni*
M034	isobrucine	33.58	0.80	*Semen Strychni*
M035	lokundjoside_qt ziziphin_qt^a^	32.82	0.76	*Semen Strychni*
M036	stigmasterol	43.83	0.76	*Semen Strychni*, *Syringa Oblata*
M037	vomicine	47.56	0.65	*Semen Strychni*
M038	ziziphin_qt^a^	66.95	0.62	*Semen Strychni*
M039	icaride A	48.74	0.43	*Semen Strychni*
M040	brucine N-oxide	52.63	0.38	*Semen Strychni*
M041	(2 R)-naringenin	42.36	0.21	*Semen Strychni*
M042	brucine	7.60	0.41	*Semen Strychni*
M043	3-oxo-tirucallic, acid	42.86	0.81	*Boswellia*
M044	alpha-boswellic acid	39.32	0.75	*Boswellia*
M045	boswellic acid	39.55	0.75	*Boswellia*
M046	tirucallol	42.12	0.75	*Boswellia*
M047	11-Keto-β-boswellic acid	29.82	0.74	*Boswellia*
M048	o-acetyl-α-boswellic acid	42.73	0.70	*Boswellia*
M049	acetyl-alpha-boswellic, acid	42.73	0.70	*Boswellia*
M050	phyllocladene	33.40	0.27	*Boswellia*
M051	incensole	45.59	0.22	*Boswellia*
M052	strictosamide_qt ^a^	76.30	0.76	*Syringa Oblata*
M053	Di (2-ethylhexyl) phthalate	43.59	0.35	*Syringa Oblata*
M054	eugenol	56.24	0.04	*Syringa Oblata*
M055	curcumin	4.37	0.41	*Rhizoma Alpiniae Officinarum*
M056	chrysin	22.61	0.18	*Rhizoma Alpiniae Officinarum*
M057	sitosterol	36.91	0.75	*Rhizoma Alpiniae Officinarum*
M058	clionasterol	36.91	0.75	*Rhizoma Alpiniae Officinarum*
M059	(2 S,3 R)-2-(3,4-dimethoxyphenyl)-5,7-dimethoxychroman-3-ol	51.89	0.37	*Rhizoma Alpiniae Officinarum*
M060	medicarpin	49.22	0.33	*Rhizoma Alpiniae Officinarum*
M061	butyl-2-ethylhexyl phthalate	44.52	0.22	*Rhizoma Alpiniae Officinarum*
M062	7-Methoxy-8-(2′-ethoxy-3′-hydroxy-3′-methybutyl)coumarin	40.36	0.21	*Rhizoma Alpiniae Officinarum*
M063	galangin	45.55	0.21	*Rhizoma Alpiniae Officinarum*
M064	5-methoxy-1,7-diphenyl-3-heptanone	68.29	0.20	*Rhizoma Alpiniae Officinarum*
M065	1,7-diphenyl-5-hydroxy-3-heptanone	61.90	0.18	*Rhizoma Alpiniae Officinarum*
M066	pinocembrin	46.08	0.18	*Rhizoma Alpiniae Officinarum*

^a^Aglycones of corresponding molecules.

### Drug Targeting and Network Analysis

Generally, herbal medicine contains numerous pharmacological compounds, which offer bright prospects for the control of complex diseases in a synergistic manner. To understand the underlying mechanism of such synergistic effect, it is important to search the knowledge about the therapeutic targets of drugs. Network pharmacology has undergone a rapid development in recent years and emerged as an invaluable tool for describing and analyzing complex systems in pharmacology studies^78, 79^. In this section, the network approach was applied to analyze the active compounds and their targets for the modified Yimusake formula from a network point of view. Presently, three compound-target networks, i.e. C-T related to vascular diseases (C-Tv), C-T associated with CNS disorders (C-Tc) and C-T linked to hormonal alterations (C-Th) networks were constructed and discussed in details, with purpose of exploring the action mechanism.

### C-Tv network: Vascular disease

**Figure 1:**
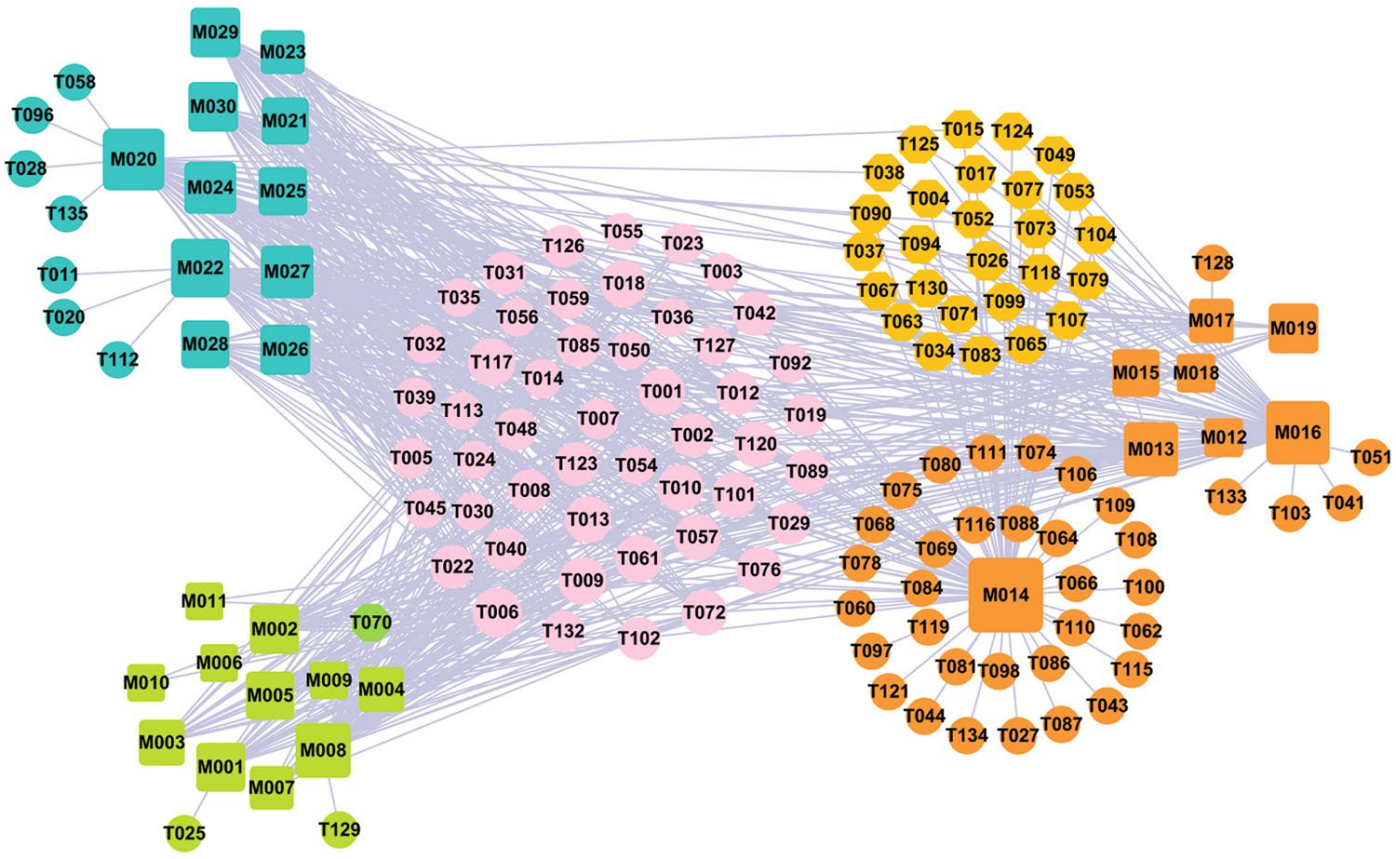
C-Tv network. 11 bioactive compounds (cyan squares) from *Myristica Semena*, 8 ones from *Stigma Croci* (dark orange squares) and 11 ones from *Gymnadenia conopsea* (yellow green squares) predicted to have 124 potential protein targets (circles). The pink circles (53) are the common targets of three herbs. The orange (27) and green (1) circles are the specific targets of *Stigma Croci* and *Gymnadenia conopsea*, respectively. Node size is proportional to its degree.

As illustrated in Fig. 1, the C-Tv network is constructed by all the active ingredients of three vascular disease-related herbs, i.e., *Gymnadenia conopsea*, *Myristica Semena* and *Stigma Croci* with their target proteins. The squares and circles represent the potential compounds and targets for *Myristica Semena* (cyan), *Stigma Croci* (dark orange) and *Gymnadenia conopsea* (yellow green), respectively, while pink circles show the overlapped targets between the three herbs. In addition, the orange and green circles are the specific targets of *Stigma Croci* and *Gymnadenia conopsea*, respectively.

The global view of this network shows that C-Tv network is composed of 30 candidate compounds and 124 potential targets. Among the 30 chemicals, quercetin (M014) has the largest number of target connections, followed by crocetin (M015) and safranal (M017). Of these targets, 50 targets are found shared by these three herbs, indicating that individual drugs in *Gymnadenia Conopsea*, *Myristica Semena* and *Stigma Croci* act on the same targets in a single formula, thus exerting possible synergistic therapeutic effect on ED.

Penile vascular disease is the most common cause of ED and may involve several pathophysiological mechanisms, including impaired arterial inflow, impaired smooth-muscle cavernosal relaxation, chronic ischaemia-induced increased cavernosal smooth-muscle contraction, cavernosal fibrosis, veno-occlusive dysfunction and chronic or episodic hypoxaemia^80^. Indeed, further observation of the C-Tv network shows that most of the high-degreed targets (>12) are associated with vascular disease. Taking NOS as an example, it keeps blood vessels dilated, controls blood pressure, and has numerous other vasoprotective and anti-atherosclerotic effects^81^. This confirms the reliability of our network and these high-degreed targets are major therapeutic targets in the treatment of vascular disease.

What’s more, 27 targets are only identified by *Stigma Croci*, indicating that the therapeutic mechanisms of *Stigma Croci* are different from that of *Gymnadenia Conopsea* and *Myristica Semena*. For example, T038 (progesterone) inhibited the proliferation of cultured human vascular smooth muscle cells, as induced by serum or endothelin-1^82^. The mitogenic effect of endothelin-1 and serum depends on mitogen-activated protein kinase (MAP-K) and MAP-kinase kinase activities, and these were significantly inhibited by progesterone, that also inhibited mitogen-stimulated c-fos and c-myc, downstream targets for MAP-K action^83^. Thus, progesterone is considered a cardiovascular-active sex steroid, which is able to regulate the structure and function of the blood vessels both in physiological as well as in pathological conditions^83^. From these results we can see that the *Stigma Croci* has unique role in activating blood circulation to dissipate blood stasis for treating vascular disease.

In addition to the common targets mentioned above, M014 (quercetin) has its own 31 targets, showing the extensively effects. Quercetin is one of the flavonoids with broad spectrum of pharmacological properties and has been used for the treatment of allergy, inflammation, arrhythmia, tumors, etc^84^. Its biological properties were consistent with its protective role in the cardiovascular system^85^. Experimental evidence indicated that quercetin exhibited an array of biological effects, such as inhibition of low-density lipoprotein oxidation, and promoted relaxation of cardiovascular smooth muscle (antihypertensive, antiarrhythmic effects), prevention of platelet aggregation (anti-thrombin (T006) effects), reduction of serum total cholesterol, and modification of the ischemia-reperfusion injury^85^. In addition, several *in vitro* studies indicated that quercetin had multiple effects on cancer cells, including the inhibition of cell proliferation and migration as well as down-regulation of expression of several heat-shock proteins^85^. Quercetin also had virucidal activity against enveloped viruses such as herpes simplex type I, respiratory syncytial, pseudorabies and parainfluenza type 3^86^. These results provide a potential mechanism that explains the multiple activities of quercetin. Combined, they suggest that the three herbs interact with diverse targets relevant with vascular disease, and thus are helpful for treating ED.

### C-Tc network: CNS disease

**Figure 2:**
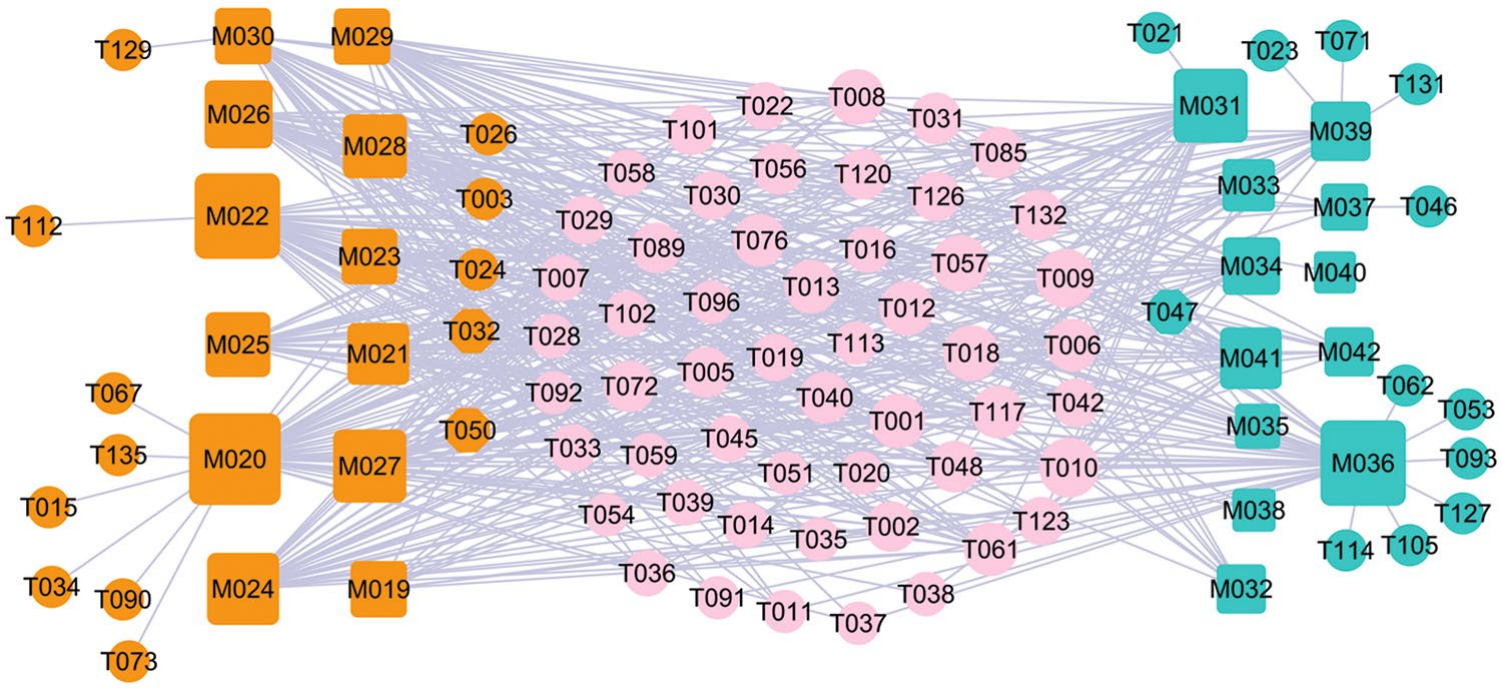
The global view of C-Tc network for *Semen Strychni* and *Myristica Semena* which is soly related to the CNS diseases. The net is composed of 11 bioactive compounds (cyan squares) from *Myristica Semena* and 12 ones from *Stigma Croci* (orange squares), as well as 78 potential protein targets (circles) these compounds interact with. The pink circles (53) are the common targets of both herbs. Node size is proportional to its degree.

Figure 2 shows the global view of C-Tc network, which is generated by 24 herbal ingredients of two CNS disease-related herbs, i.e., *Semen Strychni* and *Myristica Semena*, with their corresponding 78 potential targets. The squares and circles represent the potential compounds and their targets for *Myristica Semena* (cyan) and *Semen Strychni* (orange), respectively, while pink circles show the overlapped targets between the two herbs.

Through the analysis of the resultant network, we found three interesting phenomena. Firstly, the feature of this formula is polypharmacology. To quantify the polypharmacological effect, we counted the number of targets for each drug, that is, the degree for each drug node in the drug-target network. Network analysis results show that the average number of targets per compound is 7.78. Nearly two thirds compounds are linked with more than one target, showing the polypharmacology of the two herbs. The good hit rate indicates the rationality and reliability to find active compounds by using network-based analytical methods.

Secondly, most of the targets are associated with CNS diseases, demonstrating that the active compounds of the both herbs may cure ED through interacting with those proteins that are related to the CNS diseases. For example, as a multifunctional protease, thrombin/PAR (T006) signaling can modulate the viability of both astrocytes and neurons and has been directly implicated in several CNS diseases including ED^87^. Moreover, thrombin can also be synthesized *in situ* within the CNS, and its precursor, prothrombin, has been demonstrated in a wide range of CNS tissues^88^.

Thirdly, 53 targets (68.83%) can be commonly modulated by *Semen Strychni* and *Myristica Semena*, and multiple drugs in both herbs can also act on different targets in treating ED, indicating that both herbs may exert possible synergistic therapeutic effect on ED. For instance, brucine (M042) significantly reduced both the acetic acid-induced vascular permeability and the content of 5-hydroxytryptamine (T033) in Freund’s complete adjuvant (FCA)-induced arthritis rat’s blood plasma^89^. It is also reported that brucine inhibited the VEGF-induced neovascularization and downstream protein kinases of vascular endothelial growth factor, and then reduced the production of VEGF, nitric oxide (NO, T001)^89^. This indicates that drugs in both herbs can also act on different targets in related pathways. Therefore, it can be deduced that *Semen Strychni* and *Myristica Semena* systematically act on CNS via potentially synergistic interactions of the active compounds.

### C-Th network: Hormone-related diseases

**Figure 3:**
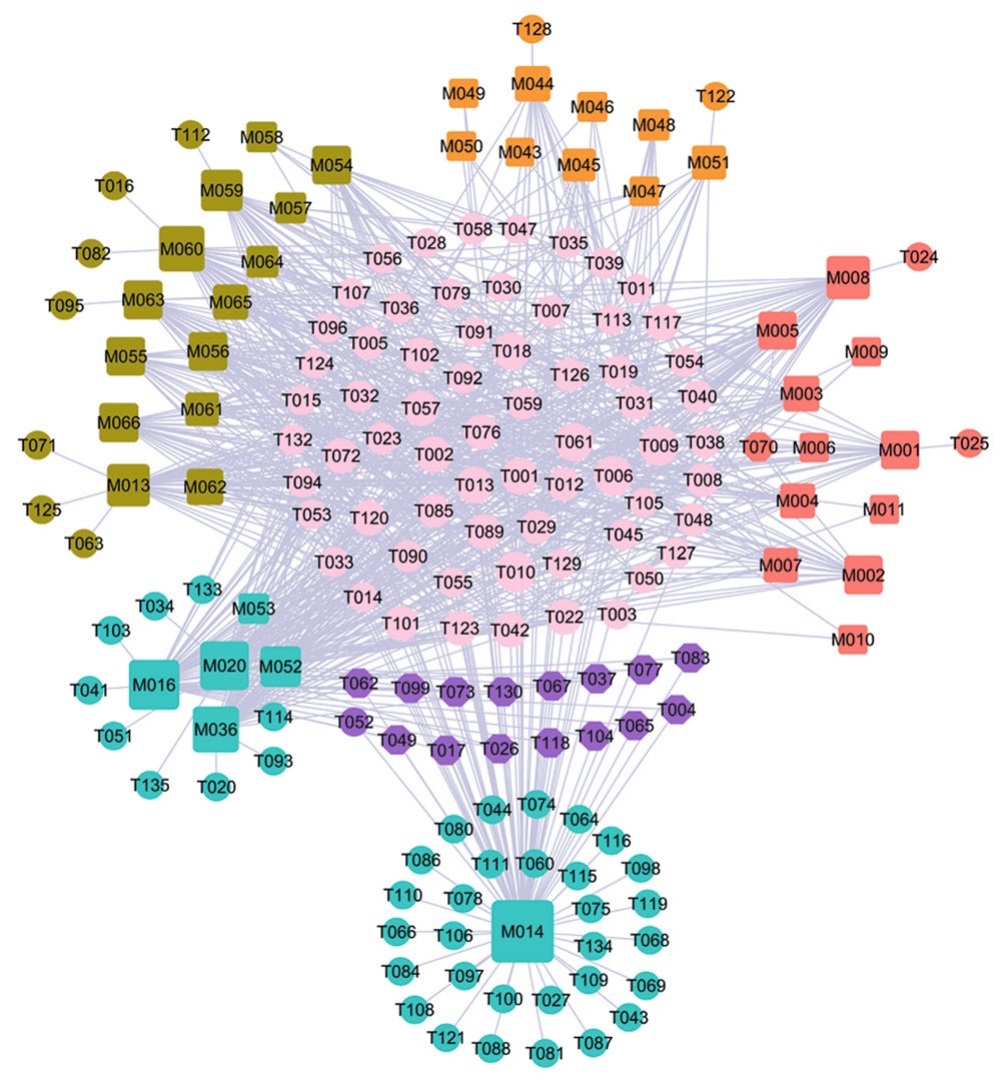
C-Tv network. The net is composed of 7 bioactive compounds from *Syringa oblata* (cyan squares), 9 ones from *Boswellia* (orange squares), 15 ones from *Rhizoma Alpiniae Officinarum* (olive squares) and 11 ones from *Gymnadenia conopsea* (red squares) as well as the 132 potential protein targets (circles) that these compounds interact with. The pink circles (53) are the common targets of four herbs. The purple circles are the specific targets of *Syringa oblata*. Node size is proportional to its degree.

Hormones are essential chemical mediators that are involved in various physiological functions, such as the sexual function of a living organism. The increase in incidence of ED with age and the progressive decrease in androgen levels in mid to late adulthood are well documented, proving the crucial roles of hormones in both the maintenance of libido and the regulation of erectile capacity in man. In view of this, a total of 132 potential targets are used to construct the C-Th network for four hormone-related diseases herbs, i.e. *Gymnadenia conopsea*, *Rhizoma Alpiniae Officinarum*, *Boswellia*, *Syringa oblata*, by linking with 40 cognate compounds (Fig. 3). For these four herbs, Fig. 3 shows a global view of the bipartite graph with color-coded nodes which correspond to either drugs (squares) or target proteins (circles): compounds in *Gymnadenia conopsea* (red), compounds in *Rhizoma Alpiniae Officinarum* (olive), compounds in *Boswellia* (orange), compounds in *Syringa oblata* (cyan). The overlapped targets among these four herbs are shown in pink circles, while the purple circles are the specific targets of *Syringa oblata*. Node size is proportional to its degree.

Similar to the above two networks, 67 targets (50.76%) are overlapped for *Gymnadenia conopsea*, *Rhizoma Alpiniae Officinarum, Boswellia*, *Syringa oblata*. This suggests that the four herbs used together to treat hormone-related diseases can by acting on the same molecular target. For example, T010 (androgen receptor) with the highest compound-target interactions are modulated by 27 compounds in four herbs. T010 is essential in the maintenance of nitric oxide-mediated erectile activity and has important functions in the regulating of erectile physiology by multiple mechanisms. The lack of androgens is known to induce a decrease in the frequency of sexual desire, sexual fantasies and sexual intercourse^90^. Also, T009 (Estrogen receptor, degree = 27) has the highest compound-target interactions. It is targeted by 11, 7, 5, 4 chemicals respectively in four herbs, which has a modulating effect on penile erectile pathophysiology and ED. Its activation is also needed for male fertility related to sperm production, and the defects in estrogenic action may contribute to male infertility^91^. Therefore, these highly degree targets should be treated as the crucial targets of these for herbs.

Moreover, apart from the overlapped targets, 16 specific targets are only connected to the *Syringa Oblata*, indicating that the therapeutic mechanisms of *Syringa Oblata* are different from the other three herbs. At the same time, M014, one of the active compounds of the *Syringa Oblata* in this network, also has the largest number of common and specific targets. Thus, we conclude that, *Syringa Oblata*, characterized as multi-targets, may exhibit therapeutic superiority for treating hormone-related diseases in comparison to other herbs in this network.

In the light of these results, we can see that *Gymnadenia conopsea*, *Rhizoma Alpiniae Officinarum*, *Boswellia*, *Syringa oblata* display a combination of multiple mechanisms for effective treatment for hormone-related diseases.

### Uncovering the Synergy from Network Pharmacology Level

Taken together, the above results provide some insights into the possible synergic effects of these seven herb. The promiscuous properties of its ingredients that one compound hits multiple receptors^92–94^. In addition, some compounds with high degree are involved in more than one herbs, such as M014 (involved *Croci Stigma* and *Syringa oblata*), M016 (involved *Croci Stigma, Syringa oblata* and *Rhizoma Alpiniae Officinarum*), M020 (involved *Myristica Semena*, *Syringa oblata* and *Rhizoma Alpiniae Officinarum*) and M036 (involved in *Semen Strychni* and *Syringa oblata*). In addition, there compounds act on different targets in related ED. Interesting, their degrees are much larger than the average value, and they also become highly interconnected compounds in the net. Thus, these compounds serve as the key role in treating ED. What’s more, more than half of the predicted targets are shared by the herbs in the formula, which indicates that individual active compounds in these seven herbs may act on same targets in a single formula, thus exerting synergistic therapeutic effects on ED. The above results suggest that TCM offers bright prospects for the control of Vascular, CNS and Hormone -related diseases in a synergistic manner.

### Experimental Validation

The ligand-binding assays for the key predicted drug-target interactions are performed to validate the inhibitory effects of all candidate compounds on their related targets. Table 2 summarizes the experimental results. Clearly, compounds methyllinolenate (M019), quercetin (M014), curcumin (M055), isorhamnetin (M013), galangin (M063), macelignan (M029) and kaempferol (M016) are tested in F2 inhibition assays in which they are proven as potent inhibitors. For example, at 50 μM, the highly-efficient compound quercetin directly binds to F2 and decreases the activity of F2 for 51% showing a well agreement with the C-Tv interactions in which quercetin has an array of biological effects such as the prevention of platelet aggregation (anti-thrombin (T006, F2) effects) in the treatment of ED. Also, curcumin, isorhamnetin, galangin, macelignan and kaempferol are potent compounds, which are able to inhibit F2 with an inhibition ratio of 35%, 49%, 41%, 59% and 54% at 100 μM, respectively. Moreover, methyllinolenate also inhibits F2 with an inhibition ratio of 58% at 200 μM. All the results of ligand-binding assays have good agreement with the observed C-T network.

**Table 2:** Inhibitory rate for the selected key C-T interactions.

NO.	Target Gene Name	Drug Name	Dosage (μM)	Inhibitory Rate (%)
1	F2	methyllinolenate	200	58
2	F2	kaempferol	100	54
3	F2	macelignan	100	59
4	F2	quercetin	50	51
5	F2	curcumin	100	35
6	F2	isorhamnetin	100	49
7	F2	galangin	100	41
8	MAOB	macelignan	50	23
9	MAOB	chrysin	100	49
10	MAOB	eugenol	50	62
11	MAOB	kaempferol	50	29

In addition, compounds macelignan, chrysin, eugenol and kaempferol are all tested in MAOB inhibition assays. As shown in Table 2, the highly effective compounds eugenol and chrysin bind to MAOB with the inhibition ratio of 62% and 49% at 50 μM and 100 μM, respectively. As a manner characteristic of inhibitors, eugenol and chrysin have important functions in the metabolism of neuroactive and vasoactive amines in the central nervous system and peripheral tissues. The good inhibition indicates the rationality and reliability of the C-Tv and C-Tc interactions, validating the network-based analytical methods. For compounds macelignan and kaempferol, they exert inhibitory activities against MAOB with an inhibition ratio of 23% and 29% at 50 μM, respectively. Both of them show relatively weak inhibitory activities, which is probably due to the characteristic of multi-targets and weak-binding affinities of herbal medicines. Weak-binding drugs from herbal medicines can be characterized by their high dissociation rates and transient interactions with their targets and thus have good efficacy^95^.

Overall, the experimental results are in good agreement with our theoretical predictions, demonstrating the reliability of the obtained C-T interactions and that the SysDT model can be used accurately and conveniently in assessing the action mode of C-T interactions.

### Computational Analysis of Candidate Compounds Binding to F2 and MAOB

**Figure 4:**
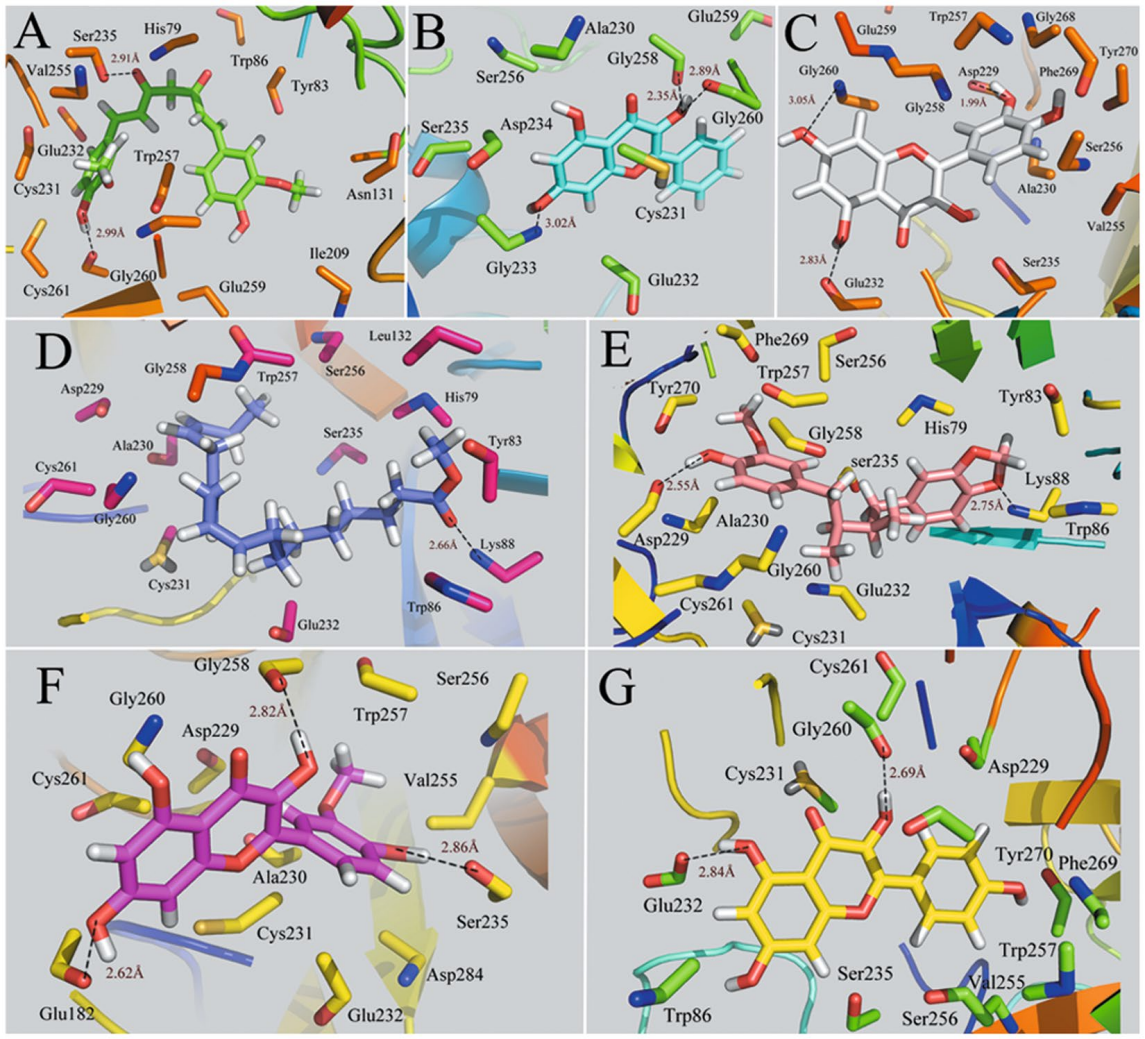
Computational modeling of F2 with their ligands. (**A**) F2-curcumin, (**B**) F2-galangin, (**C**) F2-quercetin, (**D**) F2-methyllinolenate, (**E**) F2-macelignan, (**F**) F2-isorhamnetin, (**G**) F2-kaempferol. The molecules are displayed as a ball and stick model, H-bonds are shown as dotted black lines, with distance unit of Å. Other atoms O and N are colored as red and blue, respectively.

The interacting modes of compounds methyllinolenate, kaempferol, macelignan, quercetin, curcumin, isorhamnetin and galangin with F2 receptor are depicted in Fig. 4. As seen from the figure, the -OH groups of curcumin, galangin, quercetin and kaempferol form strong hydrogen-bonds with Gly260, while the O atom of carboxyl of compounds curcumin andisorhamnetin forms one hydrogen bond with the hydroxyl group of Ser235. In addition, two hydrogen bonds are observed among compounds quercetin, macelignan and residue Asp229. These results indicate that Arg260, Ser235 and Asp239 may form a conserved hydrogen bond which acts essentially in determining the potency of the inhibitors for the binding of derivatives with F2 protein. In fact, H-bonds and hydrophobic effects, as the prominent interactions, are found in all the seven binding modes. Moreover, all the compounds have been proven to occupy approximately the same region in the F2 binding pocket. Also, the majority of H-bonds and hydrophobic modes are similar to those observed in the crystal structure of F2^96^, validating the reliability of our docking model. Taken together, the investigation of the ligand interaction modes of the docked compounds clearly demonstrates that the number and distances of H-bonds appear to play major roles in F2 inhibition.

**Figure 5:**
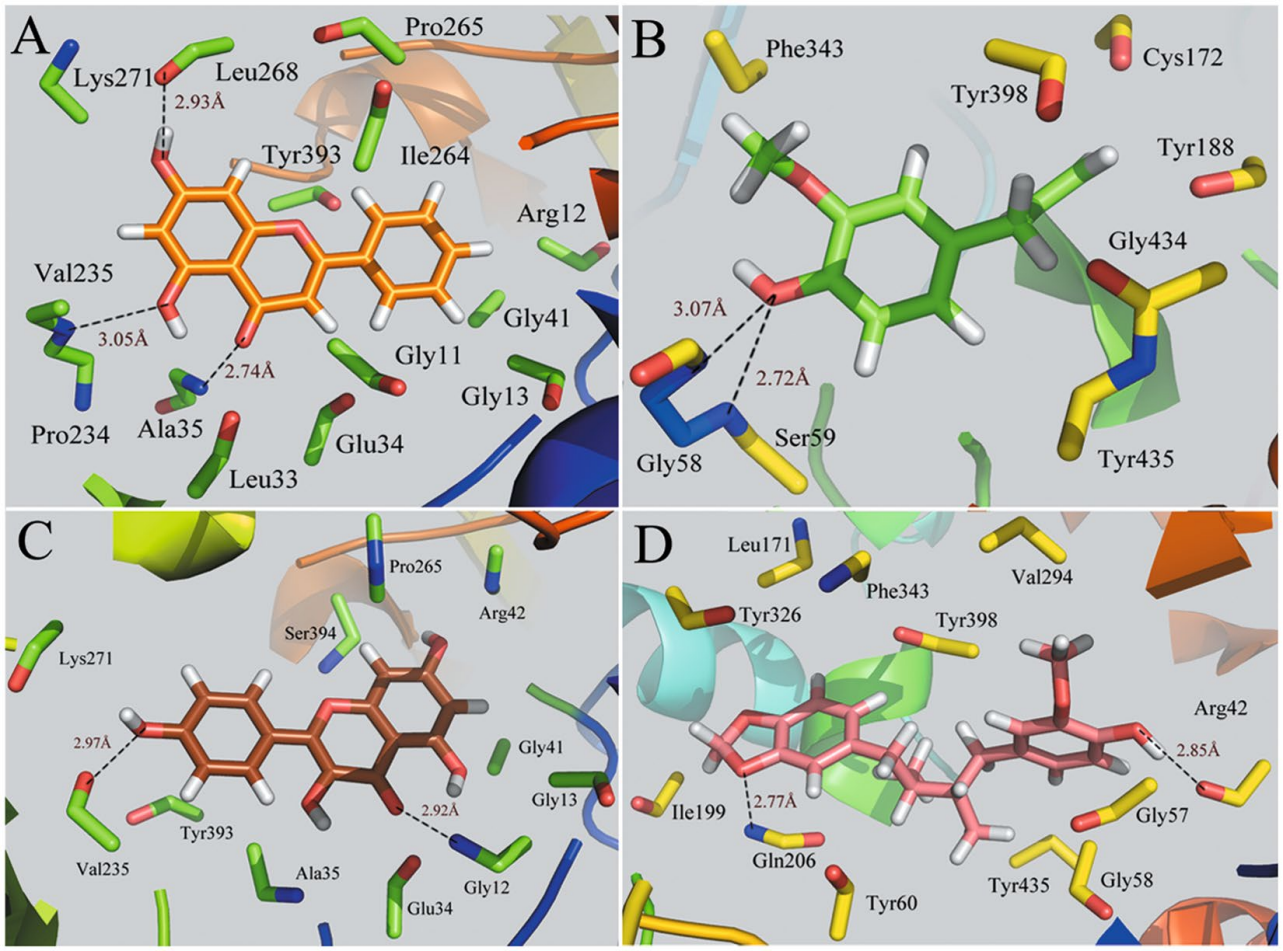
Computational modeling of MABO with their ligands. (**A**) MAOB-chrysin, (**B**) MAOB-eugenol, (**C**) MAOB-kaempferol, (**D**) MAOB-macelignan. The molecules are displayed as a ball and stick model, H-bonds are shown as dotted black lines, with distance unit of Å. Other atoms O and N are colored as red and blue, respectively.

Figure 5 shows the binding modes of complexes MAOB-chrysin, MAOB-eugenol, MAOB-kaempferol and MAOB-macelignan by using docking program. Being consistent with the experimental data, the docking simulation reveals that compound chrysin forms three H-bond interactions with residues Lys271, Val235 and Ala35 within the binding site of MAOB (Fig. 5A). As seen from Fig. 5B, eugenol is located within the substrate cavity of the enzyme, in close proximity of the flavin adenine dinucleotide (FAD) cofactor^97^. The predicted complex is stabilized by two hydrogen bonding interactions between the -OH group of eugenol and Gly58 and Ser59, explaining the best MAOB inhibitory activity of compound eugenol. Figure 5C demonstrates that kaempferol is directed towards the binding pocket in the entrance cavity, establishing hydrogen bonding interactions with residues Val235 and Gly12. As to compound macelignan (Fig. 5D), it crosses both cavities of the enzyme, locating in the piperazine nucleus between the entrance and substrate cavities separated by residues Ile199 and Tyr326^98^. Additionally, the conformation of macelignan was stabilized by two hydrogen bonds interactions with Gln206 and Arg42.

### Molecular Dynamics Simulations

It is commonly accepted that conformational flexibility and atomic-level dynamics play essential roles in facilitating protein function. When predicting the binding mode of the ligand to the receptor, molecular docking provides a good starting to evaluate the stability of the predicted interactions involved in binding. But for MD simulations, it allowed the receptor-ligand complex to be fully relaxed in the solvent environment (i.e., taking into account the protein flexibility, which cannot be fulfilled by the molecular docking process), thereby generating more reliable binding properties^99^. As a well-established method, MD simulation computationally probes the structure and dynamics of biological macromolecules. Presently, to get a dynamic picture of the conformational variations of the ligand-receptor complexes occurring in aqueous solution, the initial ligand geometries generated from the docking model on the crystal structures of F2 and MAOB in complexes with the ligands were used for MD simulations.

**Figure 6:**
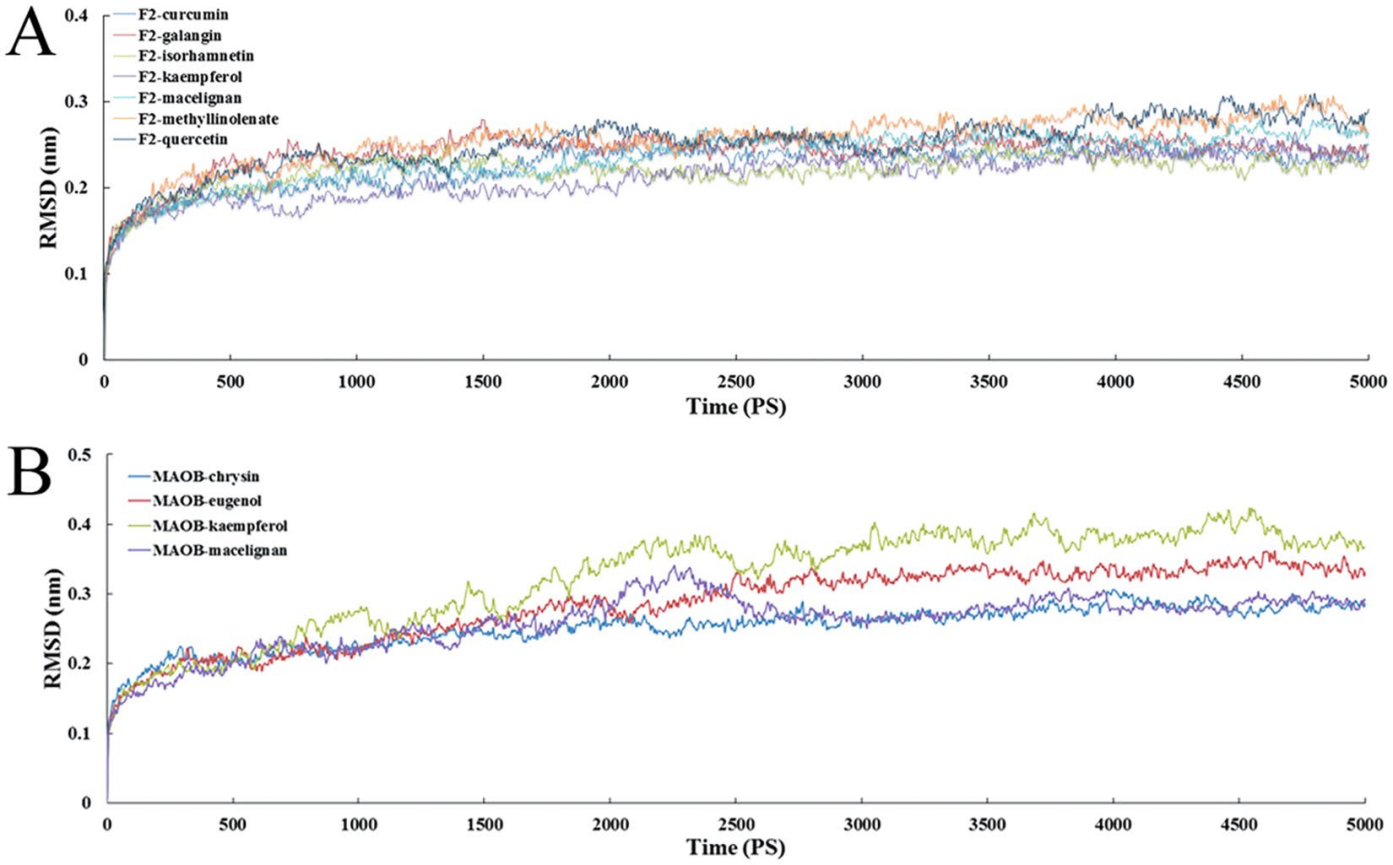
RMSD profiles of the ligands in complexes with F2 (**A**) and MAOB (**B**) for the backbone from the starting structures during the equilibration process.

In order to investigate the dynamic stability of the complexes and to ensure the rationality of the sampling method, root-mean-square deviations (RMSDs) from the starting structure were analyzed. Figure 6A and B depict the trajectories of RMSDs for the initial structure of F2 and MAOB proteins and the small ligand complexes, respectively. As depicted in Fig. 6A, after 2 ns, the RMSDs of the complex reaches about 2 Å and retains this value throughout the simulation, indicating that the overall structure of the F2 has reached a stable conformation for docked complex structure. Additionally, it can be clearly noted from Fig. 6B that the RMSDs for the four MAOB complexes reach about 2~4 Å from the beginning of MD simulation and keep this value throughout the simulation after 2.5 ns, suggesting the conformational metastability of the docked complex structure at this timescales. Overall, the RMSDs analysis confirms that the complexes reach conformational equilibrium after the simulation.

**Figure 7:**
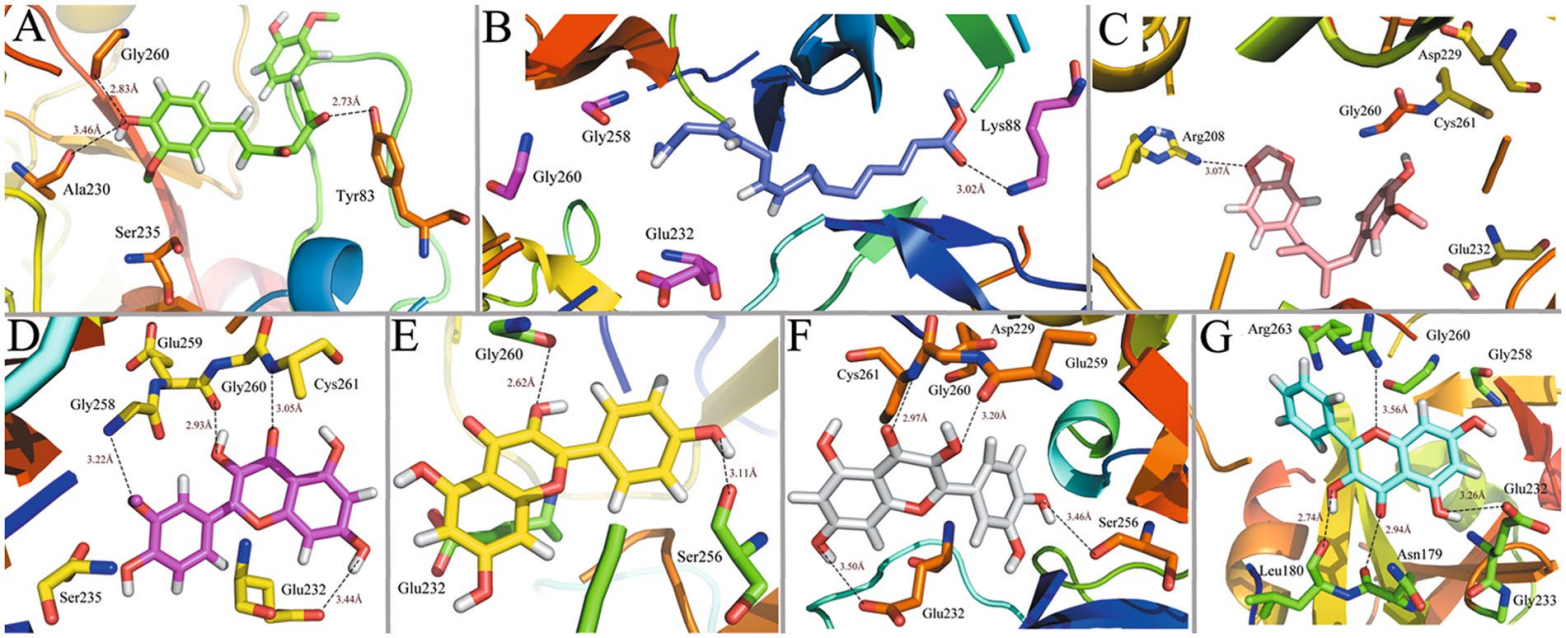
Hydrogen-bonding networks at the active site of the ligand-F2 complex from snapshots of the conformations obtained in 5-ns MD simulation for compounds (**A**) curcumin, (**B**) methyllinolenate, (**C**) macelignan, (**D**) isorhamnetin, (**E**) kaempferol, (**F**) quercetin, (**G**) galangin. The molecules are displayed as ball and stick models. H-bonds are shown as dotted black lines, with distance unit of Å. Other atoms O and N are colored as red and blue, respectively.

**Figure 8:**
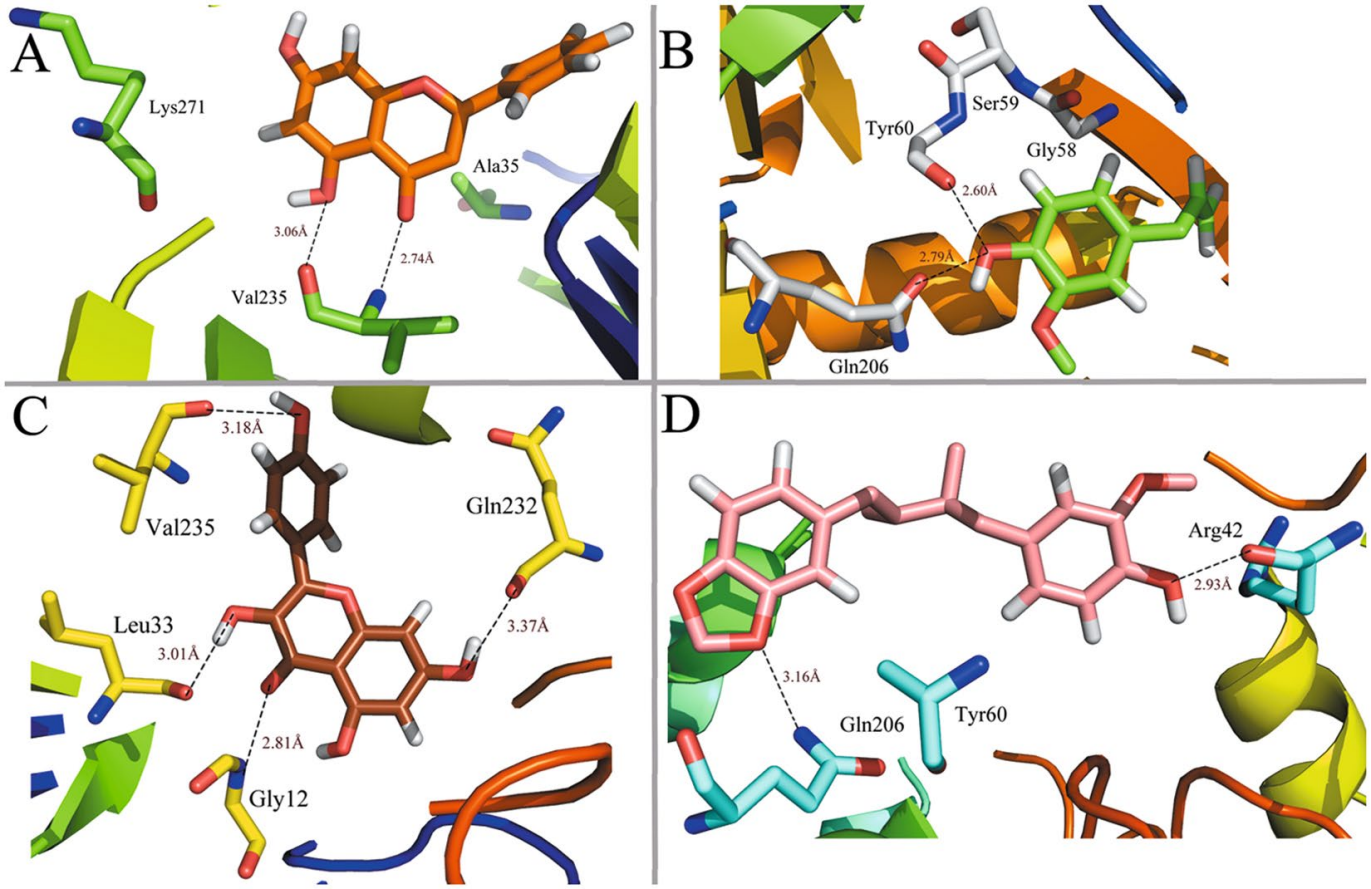
Binding conformations of different compounds in MAOB after the MD simulation. (**A**) chrysin, (**B**) eugenol, (**C**) kaempferol, (**D**) macelignan.

To probe the positional and conformational changes of the ligands related to their binding sites, the hydrogen bonding interactions were explored. Figures 7 and 8 depict the plots of the hydrogen bonding interactions including the key amino acids (within 4.5 Å distance from the ligand) of the average structure at last 1 ns of MD simulation for each complex. The binding modes of compounds methyllinolenate, kaempferol, macelignan, quercetin, curcumin, isorhamnetin and galangin with F2 receptor are depicted in Fig. 7. Obviously, Gly260 solidly forms hydrogen bonds (H-bonds) with compounds curcumin, isorhamnetin, kaempferol and quercetin, which are in good agreement with the docking results. Besides, residues Glu232 are also observed to participate in H-bond interactions with isorhamnetin, quercetin and galangin. These results confirmed that the residues Gly260 and Glu232 are highly conserved in the binding pocket and play essential roles in determining the potency of the inhibitors on their predicted protein targets. Additionally, all seven compounds have been observed to occupy approximately the same region in the F2 binding pocket and through a network of H-bonds interactions in the binding mode of F2, the ligand-receptor complex is stabilized, indicating that all compounds fit the binding pocket well.

Figure 8 shows the binding interactions of complexes MAOB-chrysin, MAOB-eugenol, MAOB-kaempferol and MAOB-macelignan after MD simulation. As illustrated in Fig. 8A, the ligand chrysin is located at the binding site by interaction with residue val235 within the receptor. Additionally, MAOB-eugenol complex is stabilized by two H-bond interactions between the -OH group of the eugenol and Tyr60 (Fig. 8B). For MAOB-kaempferol complex (Fig. 8C), it is shown that kaempferol forms two H-bonds with Gly12 andVal235 from the initial structure to the end of the simulation, which is consistent well with our previous docking results. With respect to macelignan (Fig. 8D), this compound is stabilized by two H-bond interactions between the ends of macelignan and residues Gln206, Tyr206. All these results suggest that the val235 and Gln206 are highly conserved and could be significant for the interaction for the binding of derivatives with MAOB.

Although the similarity mentioned above are obvious, the comparison between the MD and the original docking studies shows a subtle difference in the putative pocket. As shown in Figs 7 and 8, some novel H-bond interactions are formed after MD simulations, indicating the subtle changes of the conformations obtained from MD process. The reason may be that the positions of the ligands in the binding site seem undergo slight movements, which move much closer to the residues and go deeper into the pocket at the end of the MD simulation. Consequently, although the slight changes of the complexes happened in the process of MD simulation, the conformations of the ligands in the binding site is still stable, showing the rationality and reliability of our docking model.

On the basis of these interactions, the MD simulations provide an insight investigation for clarifying the mechanism of the interactions between the candidate compounds and the predicated proteins, demonstrating the potential therapeutic might effect of candidate compounds for treating ED through modulating these relevant targets and thus further validate our C-T network.

### Pathways Analysis

**Figure 9:**
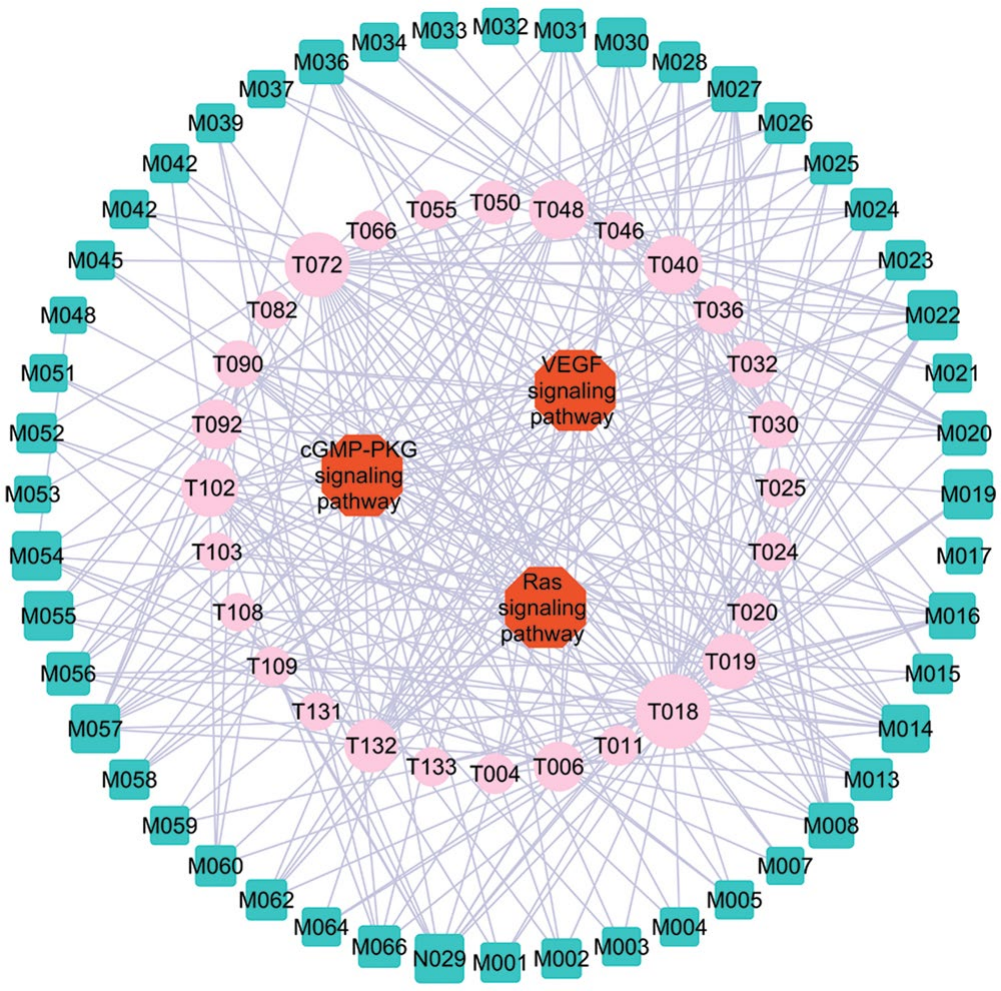
The C-T-P network was constructed by overlaying the C-T network onto T-P network. The squares and circles, respectively, represent potential compounds and targets. The pathway node is represented as red hexagon.

**Figure 10:**
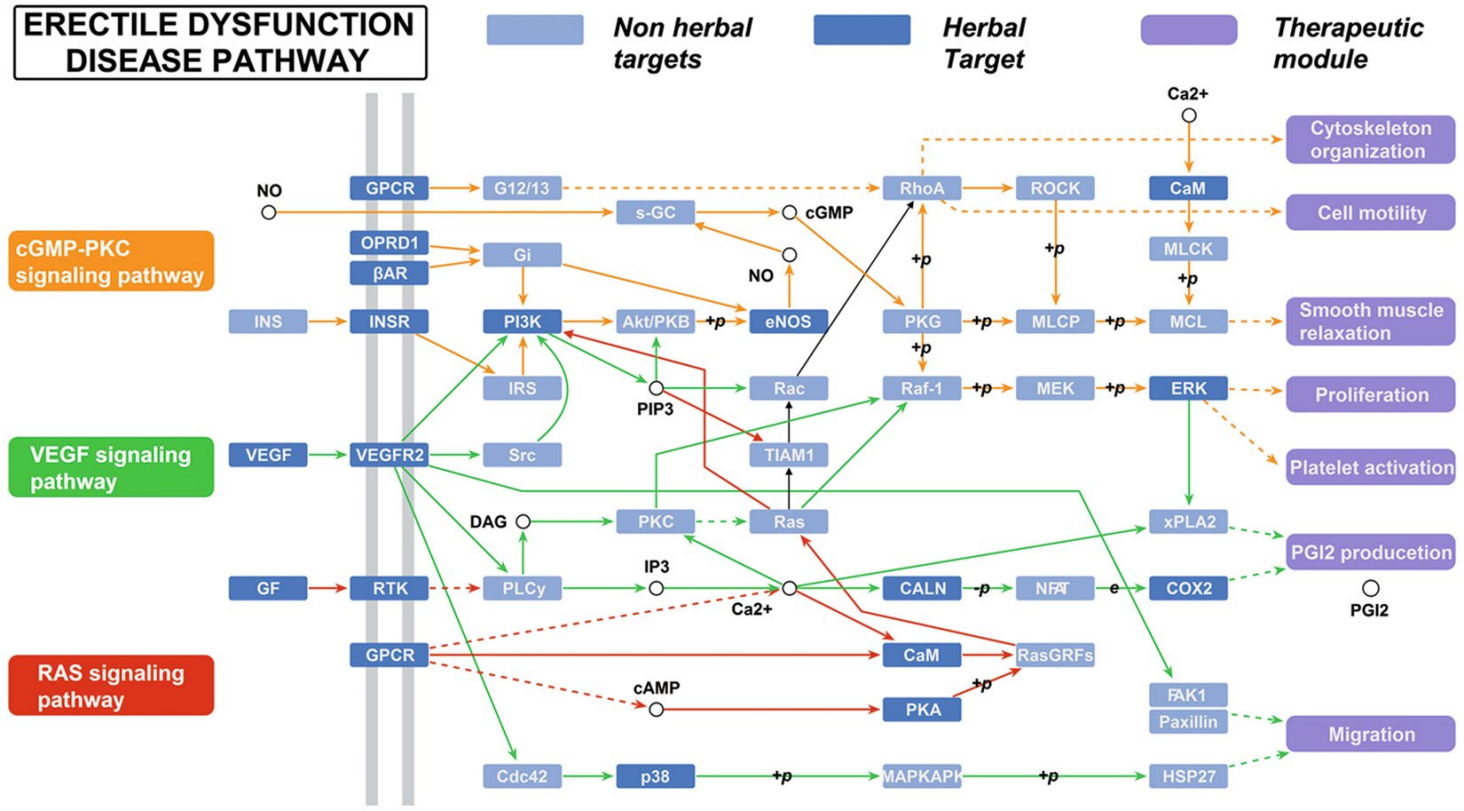
Distribution of target proteins of modified Yimusake formula on the compressed ED pathway.

For better elaborating the major pathways involved in the herbal medicines for ED therapy, the canonical pathways that are highly associated with ED were extracted from KEGG database (http://www.genome.jp/kegg/), and then all target proteins are mapped onto these pathways, resulting in a compound-target-pathway (C-T-P) network (Fig. 9). The squares and circles represent potential compounds and targets, respectively. The signal pathways are represented as red hexagons, which contain the nitric oxide (NO)/cyclic guano-sine monophosphate (cGMP) pathway, Ras kinase pathway and vascular endothelial growth factor (VEGF) pathway. As shown in Fig. 9, 28 targets of the seven herbs are linked to one or more signal systems. The three pathways were interdependent with each other through the Candidate Compounds, indicating that the modified Yimusake formula may exert synergistic influences through multiple different pathways. Additionally, a candidate compound may target different proteins involved in the same pathway or different pathways, illustrating the mechanism of multiple targets for a TCM. Moreover, to examine the systematic effects of these seven herbals on disease, an integrated “ED-related pathway” was assembled based on the current knowledge of ED disease pathology (Fig. 10).

Of all 66 candidate compounds, 27 participate in the NO/cGMP pathway, indicating that the NO/cGMP pathway plays an important role in the treatment of ED. Actually, NO/cGMP pathway is directly related to the ED, and it is a key mediator of penile smooth muscle relaxation and erection. A pharmacological approach study shows that the enhancement of NO/cGMP pathway could be necessary to treat ED^100^. In response to sexual stimulation, NO is released from nitrergic nerves and endothelia of penile arteries and trabecular tissue; it activates the guanylyl cyclase in smooth muscle cells with the production of cGMP that mediates the relaxation^101^. Either released from nerve terminals or endothelial cells, NO stimulates cGMP production in penile smooth muscle cells causing smooth muscle relaxation and increasing blood flow into the corpora cavernosac^102^. Any defect in NO/cGMP pathway at any level would result in inadequate penile smooth muscle relaxation and compromise the erectile function. Alteration of NO/cGMP signaling could compromise the relaxant capacity of erectile tissue and affect the erectile function^102^. This finding is important as it demonstrates the importance of NO/cGMP pathway for normal erectile function.

Ras signaling pathway also plays an important role in the regulation of cavernosal smooth muscle tone, the changes of which may contribute to ED in various patient subgroups, e.g. diabetes and vascular disease^103^. Presently, 27 compounds like M014 (quercetin), M016 (kaempferol) and M020 (beta-sitosterol) may disturb the Ras kinase signaling pathway through regulating its related 12 proteins like vascular endothelial growth factor receptor 2 and vascular cell adhesion protein 1, and then provide synergistic therapeutic effects to benefit patients. Indeed, recent findings have suggested an important role for Ras signaling pathway in the treatment of ED^103^. In penes, studies from several laboratories strongly support that Ras kinase-mediated smooth muscle contraction is primarily responsible for keeping the penis in a nonerect state^104^. The nerves and the endothelium of sinusoids and vessels in the penis produce and release transmitters and modulators, which interact in the control of the contractile state of the penile smooth muscles. Thus, the Ras kinase pathway participates in the regulation of cavernosal smooth muscle contraction, and changes in this pathway may contribute to various forms of ED^103^.

The third pathway, VEGF, is still one of the most commonly studied biomarkers in different diseases. Our study shows that 36 ingredients such as M001 (gymconopin B), M005 (gymconopin A) and M013 (isorhamnetin) are involved in mediating the major components of VEGF signaling pathway like prostaglandin G/H synthase 2, Nitric-oxide synthas, vascular endothelial growth factor receptor 2, which may contribute to the synergistic therapeutic effects for patients with cavernosal diseases. Many studies also confirm that the VEGF exerts its protective effects on the cavernosal tissue through endotheliotrophic, musculotrophic, and neurotrophic pathways^105^. It can also up-regulate the eNOS expression through inducing the endothelial and inducible forms of NOS in cultured endothelial cells^105^. Thus, VEGF is a potent endothelial cell mitogen to improve erectile function. These findings reveal the importance of VEGF pathway in inducing ED, which may be instrumental in the success of future human trials of new strategy to treat ED.

To sum it up, the vasodilation of penile arteries as well as the relaxation of cavernosal smooth muscle are essential processes for penile erection. These hemodynamic processes are tuned by neurovascular mechanisms that involve different ED-related pathways controlling the tone of arterial and trabecular penile smooth muscle. Alteration or disruption of one or more of these pathways could preclude the adequate relaxation of penile smooth muscle and lead to ED^106^. These pathways interact with each other through the candidate compounds, indicating that ED may exert synergistic influences on different pathways. Moreover, several protein targets belong to more than one signaling pathways, indicating that a single protein may function through multiple signaling pathways.

## Discussion

### The C-T Network from the Animal Drugs

In order to validate the reliability of the modified Yimusake formula, we have compared the results between the combinational use of the three animal components, i.e., the *Moschus*, *Ambra Grisea* and *Bullwhip materials* and modified Yimusake formula in terms of the compound-target (C-T) network interactions. A total of 37 compounds from the three animal drugs were obtained (Table 3). To further clarify the relationships between the ingredients of the Chinese herbs and their relative targets, we constructed the C-T network by connecting 32 chemicals and the protein targets. In addition, these targets were also compared with the targets of the modified Yimusake formula. The predicted targets for each bioactive molecule from the three animal components were also selected based on following principles:^31, 36^ firstly, the targets should be both presented in the RF and SVM positive prediction list (value >0.5); secondly, the targets with value of greater than 0.7 for RF and 0.8 for SVM were chosen as the final predicted targets. At last, a total of 139 targets were reserved for further analysis.

**Table 3:** Bioactive compounds of the three animal drugs, i.e., *Moschus*, *Ambra Grisea*, *Bullwhip* from the original Yimusake formula.

ID	Compound	Herb Name
S01	methyl palmitate	*Moschus*
S02	triolein	*Moschus*
S03	methyl-9-octadecenoate	*Moschus*
S04	normuscone	*Moschus*
S05	cholest-4-ene-3-one	*Moschus*
S06	Δ4-cholestenone-3	*Moschus*
S07	cholestanol	*Moschus*
S08	cholic acid	*Moschus*
S09	3α-hydroxyandrostan-4-en-17β-one	*Moschus*
S10	3α-hydroxy-5α-androstan-17-one	*Moschus*
S11	hydroxymuscopyridine A	*Moschus*
S12	androstan-4,6-dien-3,17-dione	*Moschus*
S13	androst-4-en-3,17-dione	*Moschus*
S14	5β-androstane-3,17-dione	*Moschus*
S15	5β-androstane-3α‘,17β-diol	*Moschus*
S16	5α-androstane-3β,17α-diol	*Moschus*
S17	musclide A1	*Moschus*
S18	muscopyridine	*Moschus*
S19	muscone	*Moschus*
S20	hydroxymuscopyridine B	*Moschus*
S21	3β- hydroxy-androst-5-en-17-one	*Moschus*
L01	dihydroionone	*Ambra Grisea*
L02	α-ambrinol	*Ambra Grisea*
L03	ambrein	*Ambra Grisea*
L04	(−)-a-Ambreinolide	*Ambra Grisea*
L05	(+)-amber aldehyde	*Ambra Grisea*
L06	(−)-Ambrox	*Ambra Grisea*
L07	γ-homocyclogeranyl chloride	*Ambra Grisea*
L08	epicoprosterol	*Ambra Grisea*
L09	ambrein	*Ambra Grisea*
N01	dihydrotestosterone	*Bullwhip*
N02	undecanoic acid	*Bullwhip*
N03	heptadecenoic acid	*Bullwhip*
N04	caproic acid	*Bullwhip*
N05	estradiol	*Bullwhip*
N06	testosterone	*Bullwhip*
N07	cholesterol	*Bullwhip*, *Myristicae Semena*, *Moschus*

**Figure 11:**
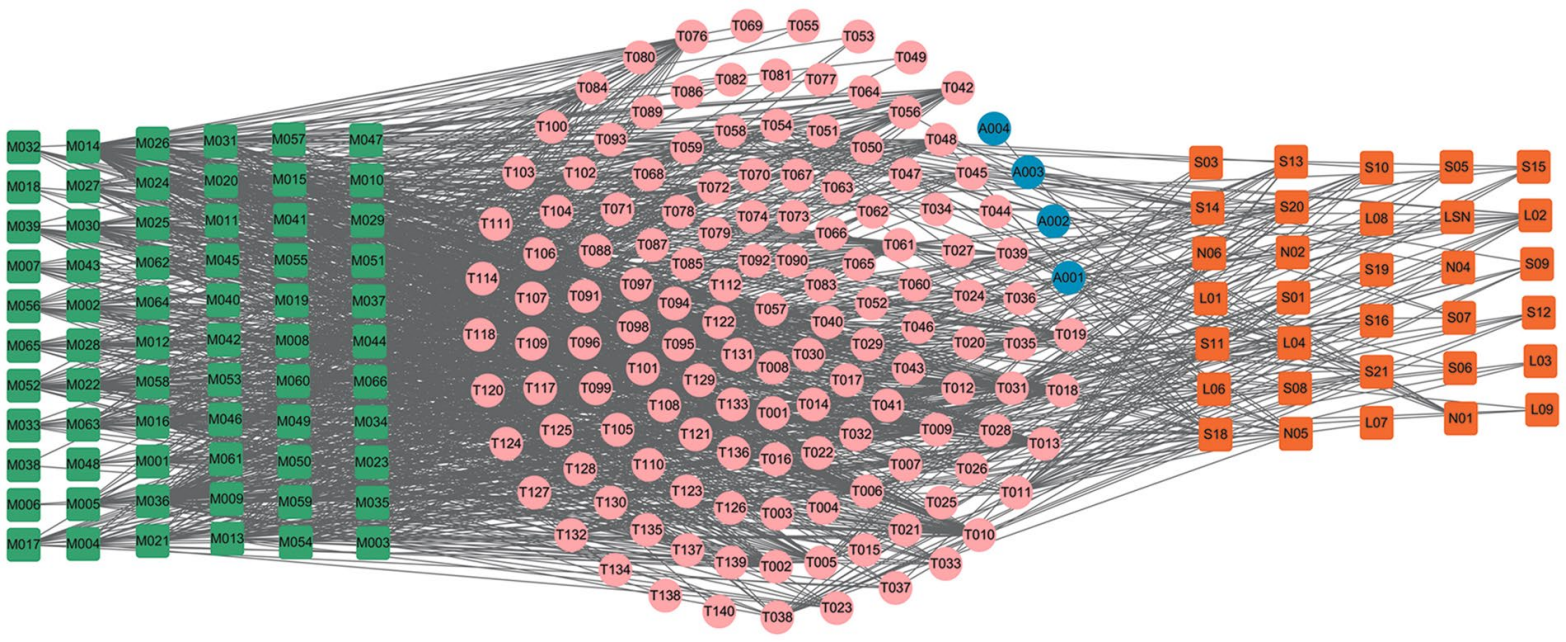
C-T network. 32 compounds (orange squares) from the three animal materials *Moschus*, *Ambra Grisea* and *Bullwhip* and 66 (cyan squares) ones from the modified Yimusake formula are connected with 139 potential protein targets (circles). The pink circles are the common targets of the three animal materials and the modified Yimusake formula. The blue circles are the specific targets of animal materials, which are not associated with ED.

As shown in Fig. 11, the C-T network is constructed by all the active ingredients of modified Yimusake formula (cyan), i.e., *Gymnadenia Conopsea*, *Stigma Croci*, *Semen Strychni*, *Boswellia*, *Semena Myristica*, *Syringa Oblata*, *Rhizoma Alpiniae Officinarum* and the three animal materials (orange), i.e., the *Moschus*, *Ambra Grisea* and *Bullwhip* with their target proteins. The squares and circles represent the potential compounds and targets, respectively, while pink circles show the overlapped targets between the modified Yimusake formula and the three animal drugs. The four blue circles are the specific targets of the three animal materials.

For most chemicals, they only link to one or two targets, while some have more than three targets. Of these targets, 97% targets are commonly modulated by the modified Yimusake formula and the three animal materials, indicating that the seven herbs contained in the modified Yimusake formula and the combination use of the three animal materials for the treatment of ED all act on very similar or mostly same molecular target. In addition, most of the targets are associated with vascular CNS and hormone diseases, demonstrating that the active compounds of the seven herbs and three animal materials may cure ED through targeting on the same proteins related to the vascular, CNS and hormone diseases. This also explains why the original Yimusake formula can treat ED by regulating the blood pressure, smooth muscle relaxation, and vasodilatation, acting on the CNS to restore penile as well as regulating the hormones levels in the maintenance of libido. What’s more, four specific targets (blue) from the animal drugs are not associated with ED. Thus, the modified Yimusake formula and three animal materials may display very similar mechanisms for effective treatment for ED, which indicates that it is possible to remove the three animal materials from this traditional medicine but still keep its efficiency.

### The C-T Network between the Original Medicine and Modified Medicine

**Figure 12:**
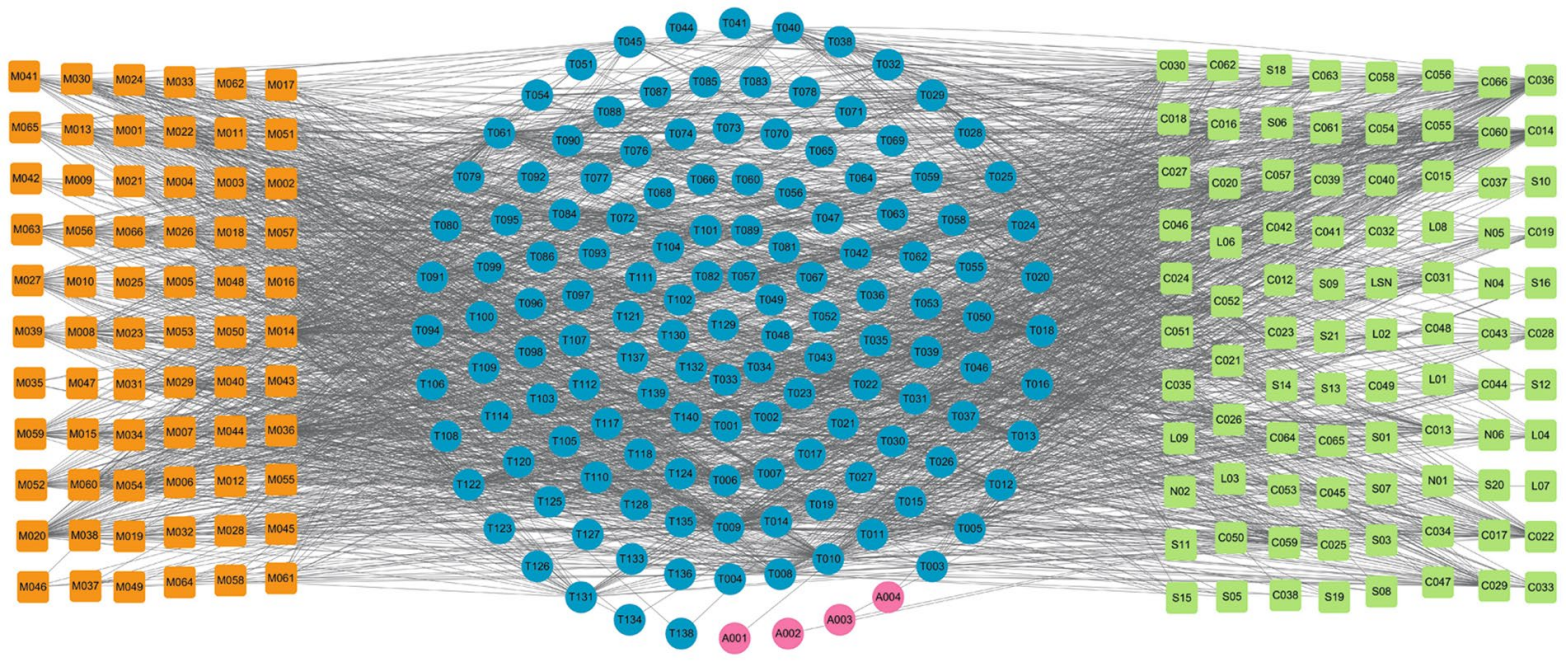
C-T network. 66 compounds (orange squares) from the modified Yimusake formula and 81 ones from original Yimusake formula (green squares) are connected with their potential protein targets (circles). The blue circles are the common targets of both formulae, while the red circles are the specific targets of original Yimusake formula, which are not associated with ED.

Additionally, in order to explore the similarity of original medicine and modified medicine, we compared the ligand-candidate target network by connecting all the active ingredients and their corresponding protein targets in the original medicine and modified formula. Firstly, we extracted all the active components of both formulae. Then, using the methods described previously, all protein targets of these compounds were predicted. The constructed C-T network is shown in Fig. 12, in which the squares and circles represent the potential compounds and targets, respectively. In this net, blue circles show the overlapped targets between the modified Yimusake formula and the original medicine, while the four red circles are the specific targets of the original medicine. Obviously, most of the target proteins are overlapped for both formulae and only four proteins are unique to the original medicine but are not associated with ED, which is consistent with the above findings. Therefore, although the original Yimusake formula has more active compounds, the modified and the original medicine also display very similar mechanisms for effective treatment for ED. This result indicates that the animal drugs may be removed from original Yimusake formula with, still, keeping its efficiency.

### May Animal Drugs Be Deleted?

Traditionally, numerous parts of animals have medicinal properties in TCM for thousands of years. So far, at least hundreds of TCM formulae contain endangered animal ingredients, like the tiger bones, horns of rhino, antelopes, cattle and goats, bovine calculi, antlers of various deer species, testicles and penis of the dog and parts of the reptile species Gecko, *et al*.^107^. However, the loss and destruction of habitats, and the excessive or uncontrolled hunting have led to a rapid reduction or even extermination of numerous animal species. As a matter of fact, many animal species used in TCM have already been listed by the Convention on International Trade in Endangered Species (CITES) of Wild Animals^108^. Medically speaking, one major negative consequence of this trend is that there will be essentially less choice for the future development of medicines. Hence, it is urgent to answer questions: Without these animal ingredients, can TCMs still keep similar therapeutic effects by some modifications? And if so, how to modify those TCMs?

As we know, animal drugs are complex mixtures containing hundreds or even thousands of different chemicals, proteins or others. Thus, to identify the active constituents of these animal materials in molecular detail through experimental methods is still intractable. Here, we propose a new strategy to analyze this formula, we called it an “alternative-removing method”. By removing the original animals, we try to investigate if the modified herbal mixture still have similar therapeutic efficacy on the disease. Through analyzing the combination effects of the whole potential active components in the formula from systematic levels, the modified formula was presented exhibiting significant correlations with ED, indicating that the modified Yimusake formula has effects in the treatment of ED.

Indeed, a famous UM formula Wenshensulapu, whose efficacy in treating ED, has recently been well established as a case study. As one of the patently and clinically approved Chinese herbal medicine prescriptions according to the new therapeutic strategy, it comprises eight plant species, i.e., *Tuber Salep*, *Croci Stigma*, *Myristica Semena,Papaver Somniferurum L*., *Rhizoma Alpiniae Officinarum*, *Aconiti Lateralis Radix Praeparata* and *Cinnanmomi Cortex*, which are very similar to our modified formula. Although there is no animal ingredient in this TCM, it still exhibited significant therapeutic effects on ED. Therefore, we assume that it is possible to modify the Yimusake formula while still keep its original curative effects after the removal of its animal ingredients. This further implies the possibility of using one or more of these herbal components as an alternative for replacement of those rare animal medicines.

The method we created not only successfully provides scientific evidence that the animal medicinal materials can be replaced in certain TCMs, but also proposes a reliable and practical strategy to systematically study the mechanism of herbal formulae and then to carry out corresponding modification process.

### Limitations

However, there are still some limitations in our study, which lies mainly in the following points. The screening platform we developed uses some filter criteria compromising the statistical average results like the DL index, which may thus produce risk of bias for the screening results. As a matter of fact, the threshold of DL 0.18 is the average similarity value of all the drug or drug candidates of DrugBank database. The use of this threshold values assumes that those compounds with DL-score no less than 0.18 may have greater potential to be a drug due to its up-above the average level of the similarity indices of the drugs of DrugBank. However, sometimes, the compounds with low DL value (<0.18) may still exhibit certain biological activities. Though this is only cases with small probability, in the future, we will still try to optimize our current methods to further improve the screening accuracy.

## Conclusion

Uyghur medicine formula, is an extracts combination of traditional Chinese herbal medicines, which have been used as valuable TCM for the treatment of a variety of diseases, including ED. However, some Uyghur medicine formulae such as Yimusake formula often contains very rare animal medicinal materials and noxious medicinal herbs. Therefore, there is a need to find new method which can replace these rare medicinal materials. The results show that:

1. Based on systems pharmacology, 66 bioactive compounds and 140 target proteins are obtained from the modified Yimusake formula, which has been identified exhibiting significant correlations with ED. The T-P network of these herbs constructed presently demonstrates that the herbal medicines may simultaneously target several pathways like NO/cGMP, Ras kinase and VEGF signaling pathways, thereby exhibiting synergistic benefits in ED treatment.
2. The experimental results and MD simulations demonstrate the reliability of the obtained C-T interactions, indicating that the SysDT model can be used accurately and conveniently in assessing the action mode of C-T interactions.
3. Most of the targets are overlapped for both the original Yimusake formulae and its modified version, and only four proteins are unique for the original formulae but are not associated with ED, indicating that the modified and the original medicines display very similar mechanisms for effective treatment of ED. The results indicate that the animal drugs may be removed from original Yimusake formula with, still, keeping its efficiency.

Overall, our study successfully provides an alternative approach to remove the animal species from TCM and explains the underlying mechanism between ED and the possible causes like vascular, neurologic and hormonal abnormalities, showing the therapeutic effects of the modified formula of Yimusake on treating ED. Also, this work gives a good example for optimizing the original TCM recipe, which is beneficial for drug development and applications.
